# Accurate *de novo* transcription unit annotation from run-on and sequencing data

**DOI:** 10.1371/journal.pcbi.1014559

**Published:** 2026-08-03

**Authors:** Paul R. Munn, Jay Chia, Charles G. Danko

**Affiliations:** Baker Institute for Animal Health, College of Veterinary Medicine, Cornell University, Ithaca, New York, United States of America; Penn State University: The Pennsylvania State University, UNITED STATES OF AMERICA

## Abstract

Functional element annotations are critical tools used to provide insight into the molecular processes governing cell development, differentiation, and disease. Run-on and sequencing assays measure the production of nascent RNAs and can provide an effective data source for discovering functional elements. However, the accurate inference of functional elements from run-on sequencing data remains an open problem because the signal is noisy and challenging to model. Here we investigated computational approaches that convert run-on and sequencing data into annotations representing transcription units, including genes and non-coding RNAs. We developed a convolutional neural network, called convolutional discovery of gene anatomy using PRO-seq (CGAP), trained to identify different anatomical features of a transcription unit, which were then stitched together into transcript annotations using a hidden Markov model (HMM). Comparison with existing methods showed a significant performance improvement using our novel CGAP-HMM approach. We developed a voting system that ensembles the top three annotation strategies, resulting in large and significant improvements in transcription unit annotation accuracy over the best performing individual method. Finally, we also explore a conditional generative adversarial network (cGAN) as a possible alternative approach to transcription unit annotation. Collectively our work provides novel tools for *de novo* transcription unit annotation from run-on and sequencing data that are accurate enough to be useful in many applications.

## Author summary

Understanding how transcriptional elements (e.g., genes) are organized and expressed is fundamental to biology and medicine. Our DNA contains thousands of transcriptional elements, but pinpointing exactly where each begins and ends (a process called genome annotation) remains technically challenging, especially for newly sequenced organisms. One powerful experimental approach, called precision nuclear run-on and sequencing (PRO-seq), captures RNA polymerase (the molecular machine that reads DNA to produce RNA) as it works across the genome, generating a characteristic “signal fingerprint” at each active transcriptional element.

In this study, we developed new computational methods that read these fingerprints more accurately than existing tools. Our main approach, called CGAP-HMM, pairs a convolutional neural network with a statistical model to identify the boundaries of active transcription units. The neural network learns to recognize distinct shapes in the PRO-seq signal corresponding to where elements start, where they end, and what happens in between. We also explored a generative AI approach (a conditional generative adversarial network) that showed promise but underperformed CGAP-HMM in most benchmarks. Combining CGAP-HMM with two established methods into a voting ensemble produced the most accurate annotations overall. These tools will be particularly valuable for annotating genomes of non-human species where reference databases are limited.

## 1 Introduction

Genomes encode a diverse collection of functional elements that provide the instruction manual for cell development, differentiation, and homeostasis [1,2]. Annotating the location and conditional activity of functional elements that make up this instruction manual is a critical goal of modern genetics. Genome annotations are tools that are crucial for understanding, cloning, and mapping genome sequences. In humans and mice, protein-coding and non-coding mRNAs are annotated through painstaking efforts of large consortia, such as RefSeq [3] and GENCODE [4] or built using population-scale RNA-seq data [5]. In addition to mRNA encoding genes, a wide variety of non-coding functional elements also serve important roles. Non-coding functional elements in the human and mouse genomes have been annotated by the concerted efforts of the ENCODE and Epigenome Roadmap consortia, which use a combination of assays (e.g., ChIP-seq, DNase, mRNA-seq) to identify active genomic regions across numerous cell types and tissues [6,7]. Efforts are underway to extend these functional annotations to common agricultural, veterinary, and plant species [8,9]. Genome annotation efforts are likely to intensify in coming years as moonshot projects designed to sequence the entire tree of life come to fruition [10]. Yet applying the approaches pioneered in human and mouse to genomes across the tree of life remains a formidable challenge due to the resources and expertise required.

An alternative approach to the integration of multiple functional assays is to focus on a single experiment which maximizes information about genome function in a sample. RNA polymerase II (Pol II), the RNA polymerase which transcribes all protein-coding genes and most non-coding RNAs, leaves characteristic patterns that can be used to distinguish a wide variety of functional elements [11,12]. Most transcription units produced by Pol II are rapidly degraded by the nuclear exosome complex [13,14], and therefore identifying them requires genome-wide measurements that directly capture RNA polymerase, such as precision nuclear run-on and sequencing (PRO-seq) or related assays [15]. PRO-seq data, when viewed in a genome browser, has characteristic shapes that are informative of the molecular function of a particular DNA sequence. Indeed, existing approaches have been proposed using a variety of machine learning and probabilistic modelling methods to interpret distinctive patterns of Pol II with substantial success [11,16].

Existing computational approaches to interpret PRO-seq data have largely focused on the discovery of either transcription initiation regions or the boundaries of active transcription units. Here we report a machine learning strategy which identifies patterns of RNA polymerase associated with several anatomical landmarks in an active transcription unit using a multi-task convolutional neural network. By combining the output of these shapes with hidden Markov models (HMMs), we developed a general strategy that discovers the boundaries of a wide variety of transcription units. Additionally, we developed an ensemble classifier using our new CNN-HMM-based method and two existing tools, groHMM [17] and T-units [18], in such a way as to overcome weaknesses of each approach. Lastly, we explored a strategy using a conditional generative adversarial network (cGAN) to directly map PRO-seq signal to transcription unit annotations. This approach was inspired by work in image processing [19] in which cGANs were used to segment noisy street scenes into meaningful object categories (e.g., cars, people, trees, roads). We hypothesized that an analogous framework might allow a model to discover biologically meaningful states in PRO-seq data without the need for manually curated training labels. These analysis tools confirm the availability of additional shapes in PRO-seq signal that we leverage here to improve the annotation of genomes using a single experimental assay.

## 2 Results

### 2.1 A multi-class CNN can identify distinct anatomical landmarks in a transcription unit

**Fig 1:**
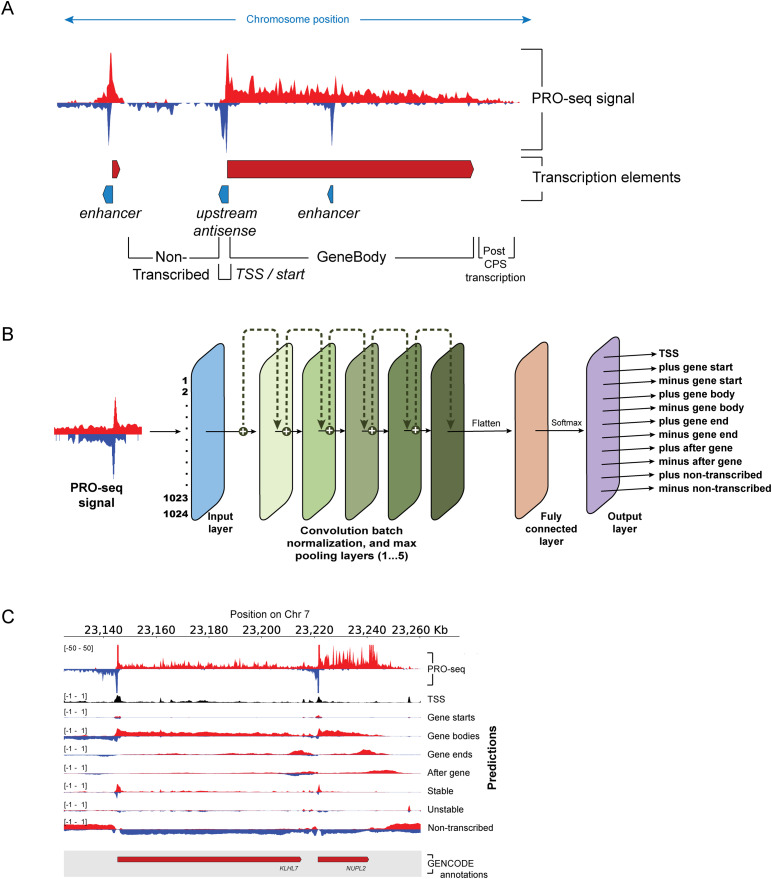
Experimental design, implementation, and performance of a convolutional neural network (CNN) designed to predict features in PRO-seq data. **(A)** Depiction of PRO-seq signal showing how the various shapes it produces correspond to different transcription element annotations. **(B)** Schematic depicting the basic structure of the CNN composed of a 1024 x 2 input layer, 5 convolutional layers, each interspersed with a batch normalization layer and a MaxPool layer, and a fully connected output layer. **(C)** An example PRO-seq signal aligned with the output CNN predictions at the *KLHL7* locus on chromosome 7. There is good correspondence between features in the PRO-seq signal and predicted features derived from the CNN.

Our first goal was to develop a strategy to discover anatomical landmarks in active genes. Motivated by the success of dREG and related tools [11], we focused on using a supervised machine learning approach to interpret patterns in PRO-seq data at multiple scales. We developed a multi-class convolutional neural network (CNN) trained to recognize the pattern of RNA polymerase associated with fifteen distinct labels, which represent different parts of the anatomy of a transcription unit. Labels include transcription start sites (TSS) which initiate new Pol II, the gene body of active transcription units, the start position of stable transcription units, the start of exosome sensitive unstable transcription units, the polyadenylation cleavage site, and transcription continuing past the polyadenylation cleavage site (Fig 1A). Since PRO-seq data provides strand-specific measurements of RNA polymerase, labels were separately enumerated on the plus and minus strand. Training examples for each label were selected using heuristics based on a variety of data, including GENCODE annotations, GRO-cap peak calls to indicate TSSs, poly-adenylation site enriched RNA-seq data to identify polyadenylation cleavage sites, and CAGE which, together with GRO-cap, separated stable and unstable TSSs (Table 1). Input to the CNN consisted of two vectors representing the PRO-seq signal on the plus and minus strand in 50 bp non-overlapping windows over a total genomic region of approximately 50 kilobases. Free parameters, including the non-overlapping window size, total genomic area provided to the CNN, and the size and depth of the CNN itself, were optimized to achieve the highest accuracy based on the area under the precision recall curve (auPRC) (see Methods).

**Table 1:** Data sources.

Data ID	Cell line	GEO	Organism	Protocol	Train/ Test	Reference
G1	K562	GSM1480327	Human	PRO-seq	Test	(Core *et al.*, 2014)
G2	K562	GSM1480325	Human	GRO-seq	Test	(Core *et al.*, 2014)
G3	K562	GSM3452725	Human	PRO-seq	Train	(Wang *et al.*, 2019)
G5	K562	GSM2361442 GSM2361443	Human	PRO-seq	Train	(Vihervaara *et al.*, 2017)
G6	K562	GSM2545324	Human	PRO-seq	Train	(Dukler *et al.*, 2017)
G7	K562	GSM2545325	Human	PRO-seq	Train	(Dukler *et al.*, 2017)
GM	GM12878	GSM1480326	Human	GRO-seq	Test	(Core *et al.*, 2014)
GH	HCT116	GSM1304424 GSM1304425	Human	GRO-seq	Test	(Allen *et al.*, 2014)
CD4	CD4	GSM2265095	Human	PRO-seq	Test	(Danko *et al.*, 2018)
MCF7	MCF-7	GSM1014637 GSM1014638 GSM1014637 GSM1014638 GSM1014639	Human	GRO-seq	Test	(Danko *et al.*, 2013; Hah *et al.*, 2011)
H1	HELA	GSE62046	Human	GRO-seq	Test	(Andersson *et al.*, 2014)
K562 GRO-cap	K562	GSM1480321	Human	GRO-cap	Train	(Core *et al.*, 2014)
GM GRO-cap	GM12878	GSM1480323	Human	GRO-cap	Test	(Core *et al.*, 2014)
DNase UW	K562	GSM736629 GSM736566	Human	DNase-seq	Train	(ENCODE Project Consortium, 2012)
DNase Duke	K562	GSM816655	Human	DNase-seq	Train	(ENCODE Project Consortium, 2012)
Poly-A	K562	xPAD database	Human	DRS	Train	(Lin et al. 2012)
GENCODE	NA	V19 Comprehensive	Human	Multiple	NA	(Harrow et al. 2012)
hg19	K562	GSE78557	Human	RNA-seq	NA	(Moore et al. 2020)
dm3	S2	GSM1032757	Fly	PRO-seq	Test	(Kwak et al. 2013)
dm3	S2	GSE145320	Fly	RNA-seq	NA	(Tchurikov et al. 2020)
mm9	HSC	GSE48895	Mouse	PRO-seq	Test	(Jonkers et al. 2014)
mm9	ES-Bruce4	GSE93453	Mouse	RNA-seq	NA	(ENCODE Project Consortium, 2012)
equCab2	Liver	PRJEB53037	Horse	RNA-seq	NA	(Peng et al. 2021)

The best performing model, which we call convolutional discovery of gene anatomy using PRO-seq (CGAP), contained an input layer of 2x 1024 windows, five convolutional and max pool layers, a fully connected layer, and an output layer of 15 nodes representing each of the pre-defined labels (Fig 1B). We trained CGAP using approximately 740,000 genomic windows and corresponding labels in K562 cells. The K562 cell line was chosen because it is an ENCODE Tier 1 line and also includes a large number of high quality PRO-seq datasets. To avoid learning features that reflect batch effects of a single PRO-seq library, we used data from four different GRO-seq and PRO-seq datasets, with two PRO-seq datasets held out as validation and test sets (see G1 through G7 in Table 1). Since the characteristic shape associated with each of the labels of interest is similar in different cell types, we assume that a model trained in K562 will be transferrable to any mammalian cell line in which features of gene and transcription unit structure are conserved.

Prior to performing empirical tests to evaluate CGAP’s predictions, we visualized data on a genome browser and observed a reasonably good correspondence between predicted labels and the expected features in the PRO-seq signal (Fig 1C). The browser showed gene body predictions increasing in signal at the transcription start site and remaining consistently high until near the polyadenylation cleavage site, at which point gene body signal trailed off as the post poly(A) signal increased. Likewise, the non-transcribed label was highest in regions with little or no signal on that strand. Predictions for non-transcribed labels were observed in regions where both gene body and gene end labels were absent, but often overlapped regions associated with unstable antisense or enhancer RNAs. The stable and unstable transcription start site states accurately marked the 5’ end of long transcription units, or short and unstable RNAs, respectively.

We evaluated the accuracy of CGAP genome-wide on held-out K562 and GM12878 datasets (average precision score for K562 = 0.76; average precision score for GM = 0.56; ROC AUC score for K562 = 0.95; ROC AUC score for GM = 0.89; S1A-S1D Fig). We note that heuristics used to define the distinct types of transcription units are an imperfect ground-truth, and therefore the estimates of genome-wide auPRC likely underestimate accuracy. Some labels performed better in K562 than GM12878. The most notable example of a cell-type difference was the label for a non-transcribed region. Since this label largely reflects a lack of signal in the surrounding region, we think cell-type differences may reflect in part the higher sequencing depth in the holdout K562 cell dataset compared with GM12878 (~400 million in K562 vs. 100 million in GM12878), as we have observed for related models [11]. Finally, the stable and unstable labels performed well in classifying whether a particular transcription start site encoded a stable transcription unit, or an unstable transcription unit that was rapidly degraded. Thus, the multi-task CNN dissected the anatomy of an active transcription unit with reasonably high accuracy.

### 2.2 CGAP predicts stable and unstable transcription at cis-regulatory elements

Existing PRO-seq-based tools such as dREG can identify cis-regulatory elements, including promoters and enhancers, with high accuracy [11,20], and our results indicate that CGAP’s TSS calls also perform well (S1A-S1B Fig). A new feature that CGAP adds beyond existing tools is the ability to classify whether a cis-regulatory element produces stable or unstable transcription [21]. This distinction is biologically meaningful: stable transcription is characteristic of protein-coding genes and lincRNAs, while elements that produce only unstable transcription are candidate enhancers that do not give rise to a stable RNA product [14,22,23].

**Fig 2:**
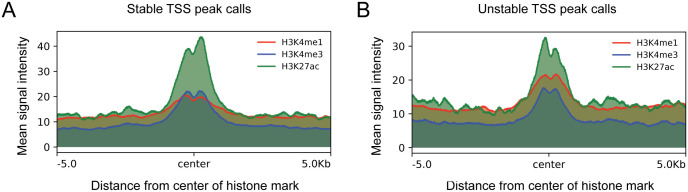
Histone mark enrichment at stable and unstable TSS peak calls. **(A)** Aggregate enrichment profiles for H3K4me1, H3K4me3, and H3K27ac centered on stable TSS peak calls. **(B)** Aggregate enrichment profiles for H3K4me1, H3K4me3, and H3K27ac centered on unstable TSS peak calls.

To provide independent confirmation of this classification, we separated CGAP TSS calls into those predicted to produce exclusively unstable transcription and those with at least one stable TSS. Stable TSSs were enriched for H3K4me3 relative to H3K4me1, while unstable TSSs showed the opposite pattern (Fig 2), reproducing the canonical chromatin signature distinguishing stable from unstable transcription [21]. These results demonstrate that CGAP’s stable and unstable classifications capture biologically meaningful differences that cannot be obtained from existing annotation tools.

### 2.3 CGAP-HMM reads the boundaries of transcription units using CGAP landmarks

We hypothesized that labels learned by CGAP representing anatomical landmarks of transcription units would be useful in improving the accuracy and sensitivity of transcription unit detection. While the predictions made by CGAP for each 50 bp region along the chromatin were reliable on average, the input PRO-seq data was extremely noisy, and CGAP predictions that did not match the surrounding signal were reasonably common (for instance, a prediction of a non-transcribed region within a gene body, or *vice versa*). Thus, CGAP alone was not able to convert predictions of these 50 bp regions into contiguous transcription units spanning many kilobases.

We reasoned that we needed an additional step to merge each 50 bp region into contiguous regions of transcribed or non-transcribed chromatin. This additional step needed to satisfy two major criteria: First, it needed to act as a smoothing function for CGAP signal, ignoring changes in CGAP state that were either small in magnitude or not sustained across broader regions. Second, recognizing that gene annotations are at best a noisy representation of the transcription occurring in any given cell type, we needed an approach that could fit robustly without a gold-standard training set.

We settled on using a hidden Markov model (HMM) for this task [24]. We compared HMMs to several discriminative methods, including a random forest classifier, an adaptive boosting classifier, a gradient boosting classifier, and a multilayer perceptron [25], across a set of metrics used to evaluate the accuracy of transcription unit boundaries as well as manual inspection of transcription unit predictions (see Methods). Not surprisingly, the decision tree-based algorithms performed worst since these models were not developed for data smoothing. The multilayer perceptron approach also severely underperformed the HMM, likely due to the unreliable training data. We did not try applying recurrent neural networks, which are more commonly used in time series classification problems, but we predict these would have similar problems to the multilayer perceptron due to a lack of gold-standard training data. We thus adopted the HMM going forward.

**Fig 3:**
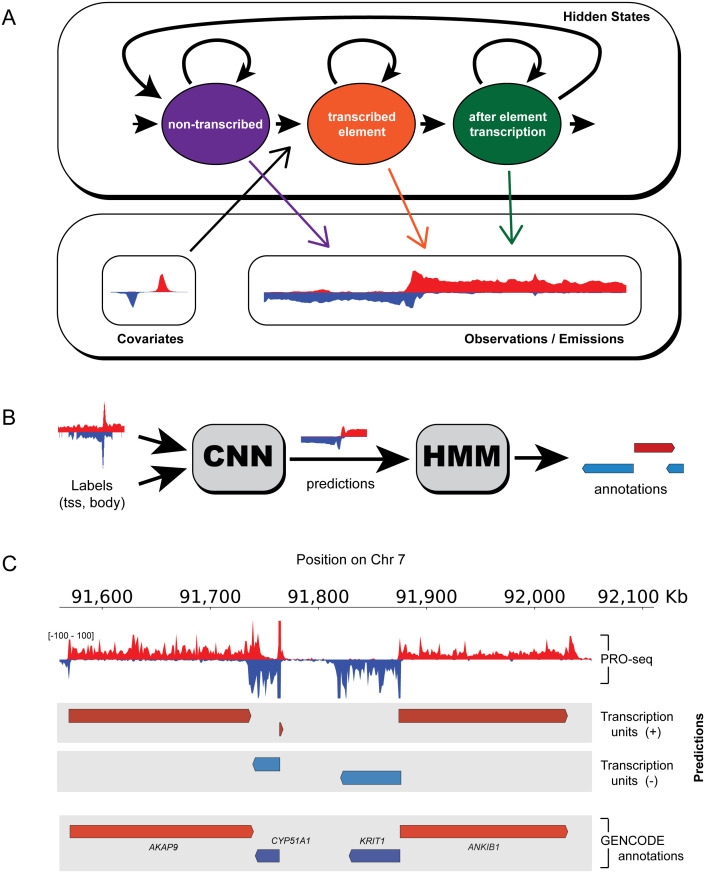
Experimental design of HMM, together with its integration with the CNN, and output predictions. **(A)** Schematic depicting the HMM configuration and allowed state transitions. Predictions made by the CNN for gene bodies are used as emissions. **(B)** Integration of the HMM with the CNN where the predictions obtained from the CNN are used as emissions and covariates for the HMM. **(C)** PRO-seq signal aligned with HMM predictions for the *ANKIB1* locus on chromosome 7 shows good correspondence between features in the PRO-seq signal and HMM predicted annotations.

The optimal HMM models PRO-seq data using three hidden states representing non-transcribed regions, gene bodies, and post-poly(A) transcription (Fig 3A). The observations supplied to the HMM are the raw continuous non-negative output values from selected CGAP tracks (Fig 3B); for each hidden state, each track was modeled independently using a gamma emission distribution, which is well suited to data that are continuous, non-negative, and right-skewed. The HMM also used plus- and minus-strand stable gene start predictions from the CNN as covariates to adjust the transition probability from the non-transcribed to the transcribed state (see Methods). We tested a range of alternative HMM structures incorporating additional CGAP labels, several of which had interesting properties but did not outperform the three-state model by our benchmarking metrics (see section 2.6).

CGAP-HMM predicted boundaries of active transcription units that agreed reasonably well with the expected patterns based on both gene annotations and manual inspection (Fig 3C).

### 2.4 Predicting transcription units using cGANs

CGAP required us to specify labels of interest and provide labeled training data. While this strategy works reasonably well in practice, it has several important limitations. First, labels are based on error-prone heuristics obtained using molecular tools that indirectly measure the quantity of interest. Second, CGAP may miss additional signal features that are highly informative about transcription unit structure but were not anticipated a priori. We therefore set out to develop a generative modeling strategy that learns informative representations of PRO-seq signal directly from the data, without the need for manually curated labels or a subsequent HMM smoothing step.

We developed a conditional generative adversarial network (cGAN) which learns to map PRO-seq signal to gene annotations. A cGAN is a generalization of a generative adversarial network (GAN) [26] which trains two separate CNNs: a generator, which generates a realistic example of data from a uniformly distributed random vector; and a discriminator, which seeks to correctly classify whether a given image was generated by the generator or is a real example of the data (see Methods). A cGAN builds on this concept by transferring specific samples from an input domain to the desired output domain. For example, cGANs introduced by [19] learn mappings from grayscale, outline pictures of handbags to fully colored images of the same bags, or learns mappings from satellite images to a street map view of the same area.

**Fig 4:**
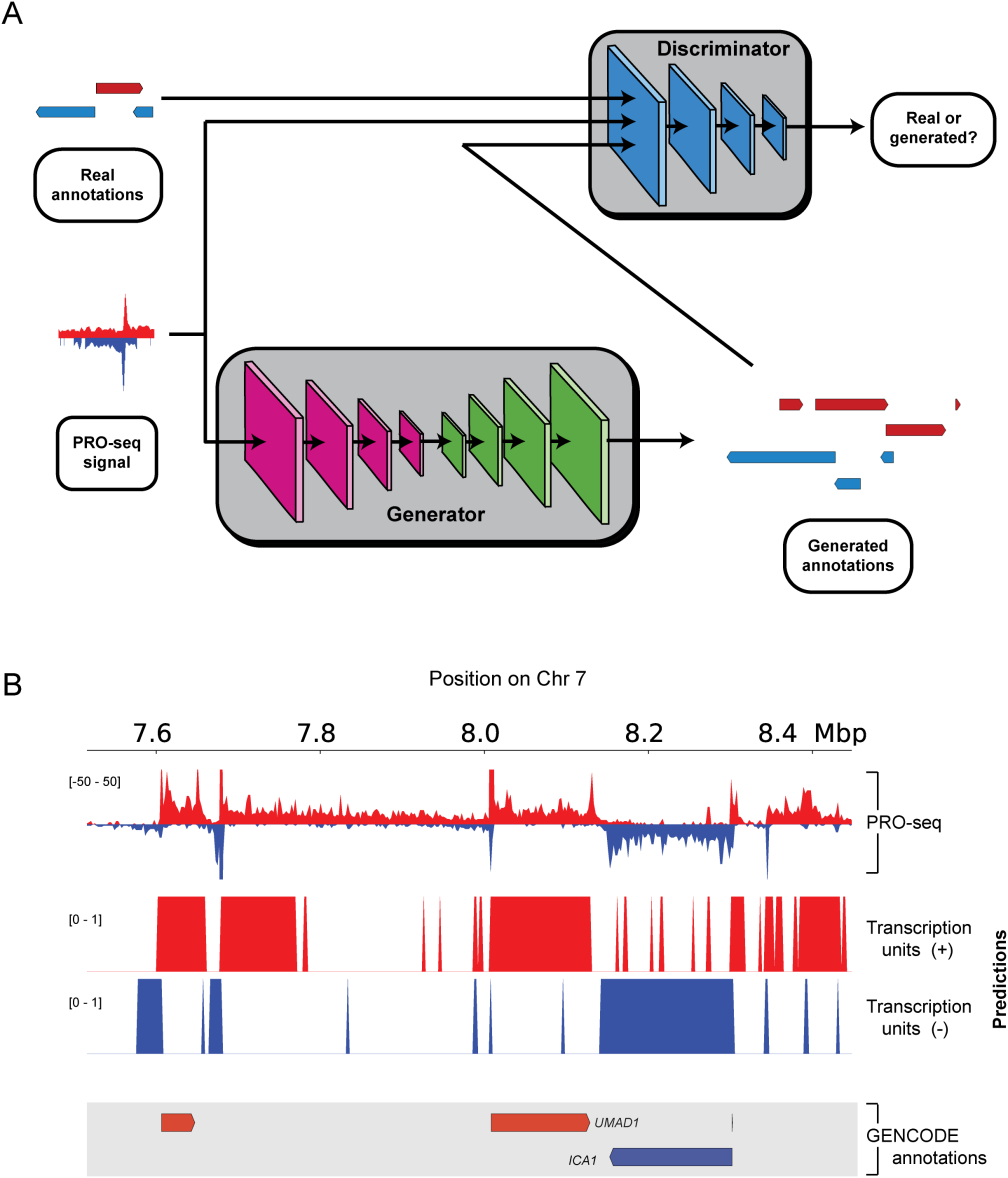
Experimental design of the cGAN and example predictions. **(A)** The cGAN is designed to produce generated annotations via a generator network (set up as a modified encoder-decoder). If trained correctly, the quality of the generated annotations should be at such a high level that, when fed into a discriminator (implemented as a PatchGAN with a similar set up to the encoder), the latter is unable to distinguish between the generated and the real/actual annotations. **(B)** Example PRO-seq signal and cGAN predictions for the *UMAD1* locus on chromosome 7 shows good correspondence with the longer, well-expressed features in the signal.

We used a cGAN to learn the mapping between PRO-seq signal and gene annotations. Our cGAN (Fig 4A) takes as input PRO-seq data in 50 bp non-overlapping windows on both the plus and minus strand over a genomic interval of 1.6 MB. We trained the cGAN to perform translations from PRO-seq signal to transcription units (Fig 4B). We attempted to use two sources of transcription units: first, the set of active GENCODE annotations, and second, annotations derived from an ensemble method combining the best three performing transcription unit annotation approaches (see Methods); both of which performed similarly in benchmarks. The trained cGAN takes as input PRO-seq data over a 1.6 MB genomic interval and returns a value between 0 and 1 representing the confidence that 50 bp bin is found inside of a gene annotation.

### 2.5 Assessing CGAP-HMM’s strengths and weaknesses compared to predictions made using RNA-seq data

RNA-seq data is currently the gold-standard for functional genomic transcript annotation in new species. To evaluate whether annotations inferred from nascent transcription provide information not captured by RNA-seq-based gene predictions, we compared CGAP-HMM predictions generated from PRO-seq data with annotations predicted by BRAKER3 [27] using RNA-seq data. This comparison asks a different question than the benchmark against other PRO-seq transcription-unit callers described below. Here, the goal was to determine whether PRO-seq provides a practical advantage for defining the boundaries and continuity of active transcription units, relative to a widely used RNA-seq-guided annotation approach. Because PRO-seq measures engaged RNA polymerase directly, we expected CGAP-HMM to perform best at identifying active initiation and continuous transcription across gene bodies. By contrast, because RNA-seq measures accumulated mature RNA, we expected BRAKER3 to perform best when the objective is to infer mature gene models, splice structure, and transcript boundaries that are well represented in steady-state RNA.

**Fig 5:**
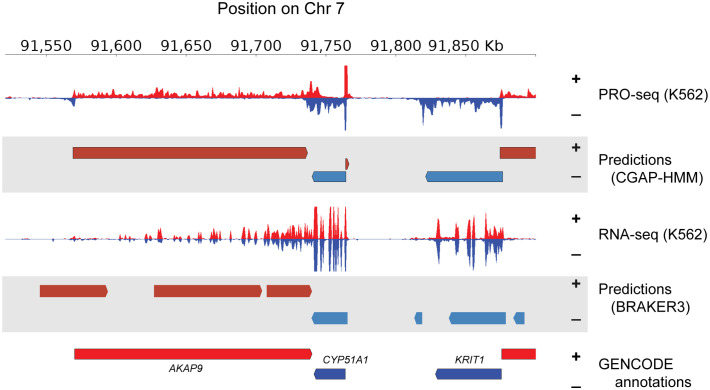
Browser-track comparison of CGAP-HMM predictions generated from PRO-seq data and BRAKER3 predictions generated from RNA-seq data at the AKAP9 locus on chromosome 7.

Browser-track comparisons at the *AKAP9* locus on chromosome 7 illustrate the qualitative differences between the two approaches (Fig 5). CGAP-HMM generally followed the broad domain of nascent transcription detected in PRO-seq and preserved the continuity of active transcription units. In contrast, BRAKER3 more frequently broke the same locus into multiple predicted annotations. This behavior is consistent with the type of evidence used by RNA-seq-guided gene prediction: RNA-seq coverage is concentrated over mature exons and splice junctions and therefore can provide strong information about exon-intron structure while providing less direct evidence for continuous nascent transcription across the full transcription unit. These browser examples suggested that CGAP-HMM and BRAKER3 recover overlapping but non-identical views of gene activity.

**Fig 6:**
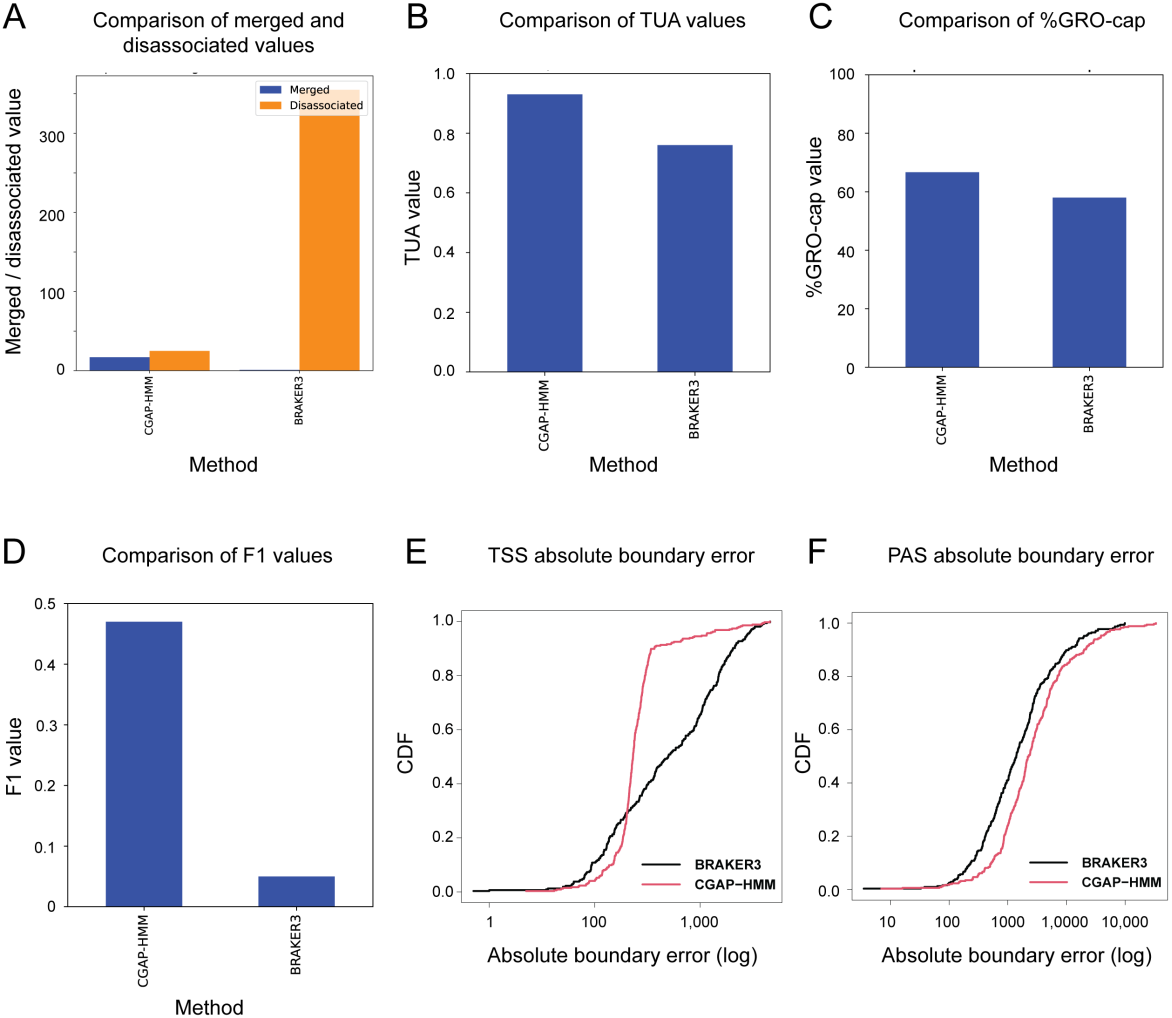
Quantitative comparison of CGAP-HMM predictions generated from PRO-seq data and BRAKER3 predictions generated from RNA-seq data. **(A)** Comparison of merged and disassociated annotations. CGAP-HMM maintains a more favorable balance, whereas BRAKER3 disassociates annotations at a higher rate. **(B)** TUA values, where higher values indicate better agreement with reference transcription-unit boundaries. **(C)** Percentage of GRO-cap sites recovered, measuring recovery of active transcription initiation sites. **(D)** F1 scores, measuring the balance between precision and sensitivity. **(E)** Cumulative distribution frequency of absolute TSS boundary errors, showing that CGAP-HMM has smaller errors and more frequently places predicted starts upstream of the reference TSS, whereas BRAKER3 more frequently places predicted starts downstream. **(F)** Cumulative distribution frequency of absolute PAS boundary errors, showing that CGAP-HMM more frequently extends predictions beyond the PAS, while BRAKER3 has smaller PAS errors.

We next quantified these differences across the evaluation set. CGAP-HMM performed well in the comparison of merged and disassociated annotations, whereas BRAKER3 disassociated annotations at a much higher rate (Fig 6A). A high disassociation rate indicates that a single reference annotation is frequently split into multiple predicted features. For transcription-unit discovery, this is an important failure mode because it can obscure the relationship between a promoter, the transcribed gene body, and the downstream termination region. Thus, CGAP-HMM better preserved the continuity of active transcription units, whereas BRAKER3 was more prone to fragmenting annotations into separate RNA-seq-supported segments.

CGAP-HMM also outperformed BRAKER3 by the TUA metric (Fig 6B). Because TUA summarizes agreement between the predicted and reference transcription-unit boundaries, higher TUA values indicate better recovery of the overall extent of the transcription unit. The stronger CGAP-HMM performance on this metric suggests that PRO-seq-derived predictions more accurately represent the span of active transcription, despite the specific weakness of CGAP-HMM at the 3’ boundary described below. CGAP-HMM also recovered a slightly higher fraction of GRO-cap sites than BRAKER3 (Fig 6C). Since GRO-cap marks active transcription initiation, this result supports the expectation that nascent transcription data are particularly informative for identifying active promoters and promoter-proximal transcription events, including features that may be weakly represented or absent in steady-state RNA-seq. Finally, CGAP-HMM achieved a higher F1 score than BRAKER3 (Fig 6D), indicating a better balance between sensitivity and precision when recovering reference annotations.

The largest advantage of CGAP-HMM was observed at the 5’ end of transcription units. CGAP-HMM produced smaller TSS boundary errors than BRAKER3, and the cumulative distribution of absolute TSS boundary errors confirmed that CGAP-HMM predictions were closer to the reference TSS (Fig 6E). The direction of the TSS errors was also informative: CGAP-HMM usually placed the start of the predicted transcription unit upstream of the annotated TSS (S2A Fig), whereas BRAKER3 more often placed the start downstream of the TSS (S2B Fig). This difference is consistent with the underlying assays. PRO-seq captures promoter-proximal RNA polymerase and therefore contains direct information about transcription initiation, while RNA-seq often lacks precise information at the 5’ end because mature RNA abundance, transcript processing, and incomplete 5’ coverage can shift evidence downstream of the true initiation site. Thus, CGAP-HMM is better suited for identifying active TSS-proximal transcription boundaries.

In contrast, CGAP-HMM performed worse than BRAKER3 at the PAS boundary. CGAP-HMM frequently extended predictions beyond the annotated PAS, resulting in larger PAS boundary errors, and the cumulative distribution of absolute PAS boundary errors showed that these errors were larger than those observed for BRAKER3 (Fig 6F). Again, the direction of the PAS errors was also informative: CGAP-HMM most often placed the end of the predicted transcription unit downstream of the annotated TSS (S2C Fig), whereas BRAKER3 more often placed the end upstream of the TSS (S2D Fig). This behavior is expected for a PRO-seq-based method because RNA polymerase often continues transcribing downstream of the polyadenylation cleavage site before termination. Although the three-state HMM explicitly models post-PAS transcription, distinguishing the mature transcript endpoint from downstream engaged polymerase remains a difficult problem. BRAKER3, by contrast, benefits from RNA-seq evidence derived from processed transcripts and therefore more accurately marks the mature 3’ end in this comparison.

Together, these results show that CGAP-HMM and BRAKER3 have complementary strengths. CGAP-HMM is preferable when the goal is to define cell-type-specific active transcription units, recover active initiation sites, preserve transcription-unit continuity, and detect promoter-proximal or unstable transcription using nascent transcription data. BRAKER3 is preferable when the goal is to construct conventional gene models from RNA-seq, especially when exon-intron structure, mature transcript isoforms, and 3’ boundary placement are the primary objectives. Therefore, the use of PRO-seq data for annotation calling is justified when the biological question concerns active transcription rather than the accumulated mature RNA population.

### 2.6 Performance comparison of CGAP-HMM, cGAN and existing transcription unit prediction tools

**Fig 7:**
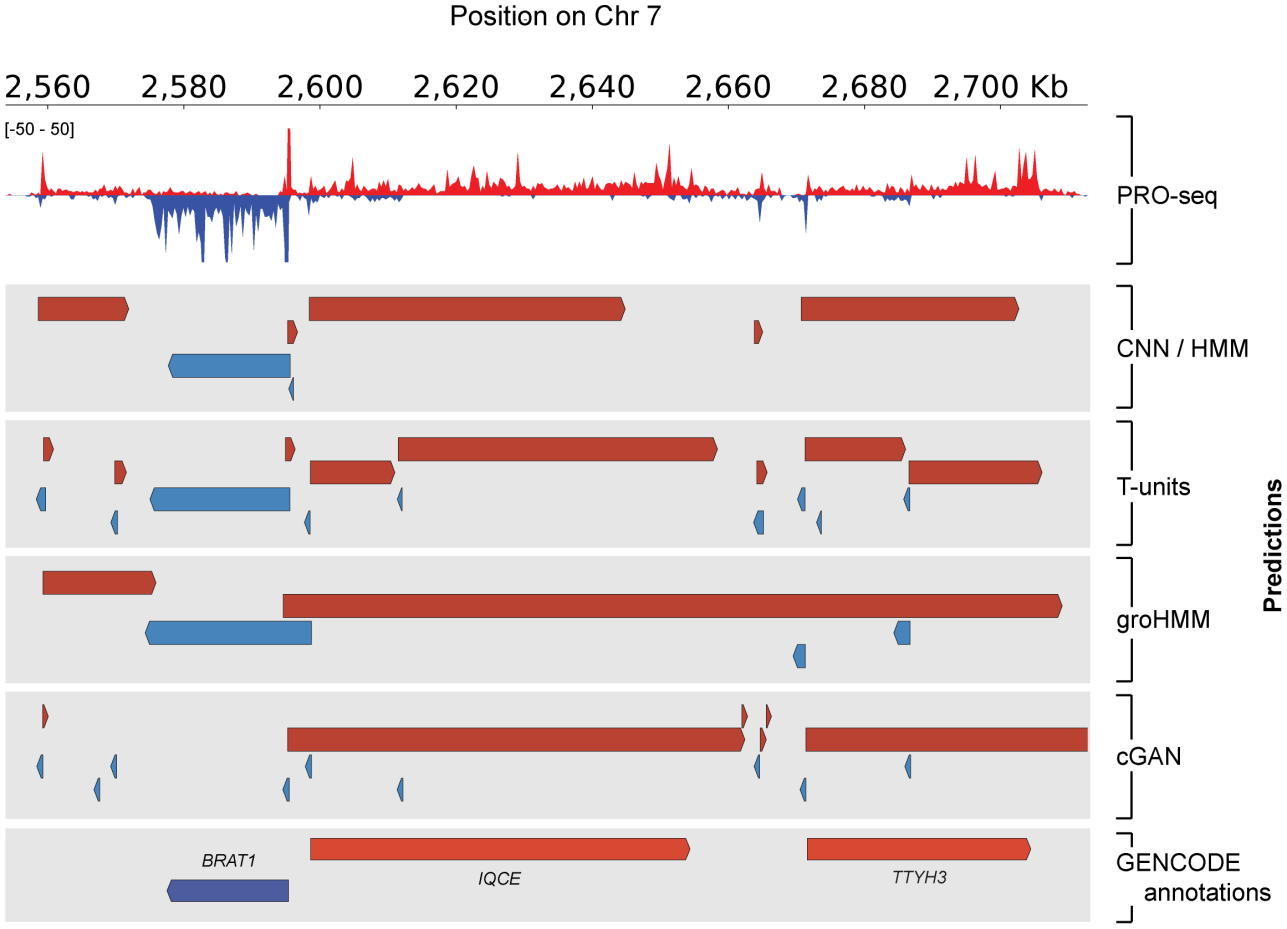
Comparison of the CNN/HMM and cGAN with other annotation methods for predicting features within PRO-seq data. T-units predictions at this locus have broken up both the IQCE and TTYH3 genes, whereas groHMM has merged both together. However, the CNN/HMM has predicted all three genes correctly, with some errors finding the PAS. The cGAN overestimates the lengths of these individual genes, fragments many of its predictions, and fails to identify transcription units on the minus strand.

**Fig 8:**
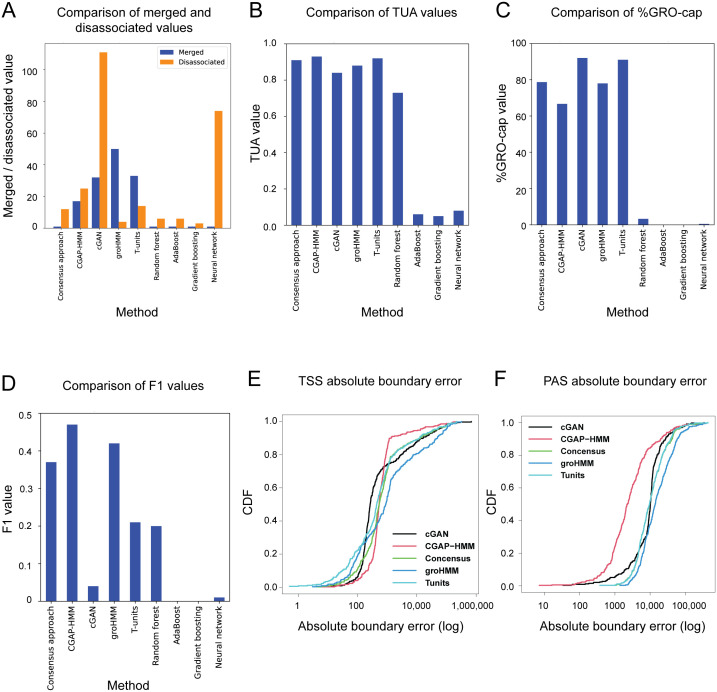
Metrics used to evaluate the performance of each of the methods. **(A)** Comparison of merged and disassociated values. **(B)** Comparison of TUA values. **(C)** Comparison of %GRO-cap values. **(D)** F1 scores, measuring the balance between precision and sensitivity. **(E)** Cumulative distribution frequency of absolute TSS boundary errors, showing that CGAP-HMM has smaller errors and more frequently places predicted starts upstream of the reference TSS. **(F)** Cumulative distribution frequency of absolute PAS boundary errors, showing that groHMM, T-units, and cGAN more frequently extend predictions beyond the PAS; CGAP-HMM also does this but has smaller PAS errors.

Using our set of high confidence GENCODE annotations (see Methods), we compared the predictions made by the CGAP-HMM and the cGAN approach to existing transcription unit identification methods by manual inspection of the UCSC genome-browser. We selected two methods (groHMM [17] and T-units (Danko et al., 2018, GitHub: https://github.com/andrelmartins/tunits/tree/master)) that use nuclear run-on assays (GRO-seq and PRO-seq respectively) to identify the boundaries of transcription units. Both T-units and groHMM use similar HMMs on raw PRO-seq to identify transcription units. groHMM was introduced using a two-state HMM, dividing the genome into transcribed and non-transcribed regions. T-units improved on groHMM by adding a third state that attempts to model transcription occurring after the end of a transcription unit, and by making the transition from the non-transcribed to the transcribed state conditional on TSSs detected using dREG. Comparison of transcription unit calls on a genome browser demonstrate the strengths and weaknesses of the distinct methods (Fig 7A). T-units fragments its predicted annotations at a higher rate than CGAP-HMM whereas groHMM merges predicted annotations together at a higher rate compared to CGAP-HMM (Figs 7A and 8A). Both T-units and groHMM frequently over-estimate the length of genes, incorporating post CPS transcription into the gene estimate (Figs 7A and 8F). Nevertheless, all three transcription unit annotations performed reasonably well at most loci. In contrast, the cGAN approach split the majority of annotations into multiple parts, both overestimating or underestimating the length of transcription units (Figs 7A, 8A, and 8F).

The differences in the performance of the CGAP-HMM, T-units, and groHMM methods provided us with the motivation to combine the three in such a way as to maintain the strengths of each while also attempting to compensate for their different weaknesses (described in Methods, section 4.9 *Building a consensus for CGAP-HMM, groHMM, and T-units*).

We evaluated this consensus approach and all individual methods (including each of the decision tree-based and neural network approaches mentioned above in section 2.3 *CGAP-HMM reads the boundaries of transcription units using CGAP landmarks*) using several types of metrics that measure different aspects of transcription unit identification. First, we compared the rates at which each method disassociated gene annotations into separate candidate transcription units, and the rate at which separate genes were merged into a single candidate transcription unit annotation (see Methods, section 4.10 *Accuracy metrics for transcription unit identification*; S4A-S4B Fig). We note that while the optimal method would get both values as low as possible, incorrect parameter tuning can result in one value decreasing at the expense of the other. For example, it would be easy to decrease the count of annotations disassociated by increasing the number of annotations merged (or vice versa), but this does not provide a good solution to the transcription unit identification problem. CGAP-HMM performed the best on this metric, with a relatively even balance between merged and disassociated transcripts, compared to T-units and groHMM’s low disassociation rate but much higher merge rate. The cGAN did not perform well in this metric, merging fewer transcripts than groHMM, but disassociating significantly many more (Fig 8A). It should be noted that the decision tree-based and neural network approaches appeared to perform exceptionally well on this metric, but this result is misleading since these methods predicted so few annotations that the number that were merged and/ or disassociated is artificially low.

Next, we examined the TUA metric [17], which measures the accuracy of the 5’ and 3’ end of transcription unit predictions (Fig 8B). The TUA metric ranges between 0 and 1, and values near 1 indicate higher agreement with the ground truth. A comparison of TUA showed that CGAP-HMM and T-units both achieved the best results, a TUA of 0.93 and 0.92 respectively, higher than groHMM. The cGAN did not perform well in this metric, although it outperformed the decision tree-based and neural network approaches (Fig 8B).

As a metric for how different TU callers handled unstable, intergenic transcripts, we examined the fraction of intergenic GRO-cap sites that were captured by each transcription unit prediction method. cGAN performed with the highest sensitivity in this task, discovering 92% of GRO-cap sites, and T-units was close behind. CGAP-HMM performed well with 67%, and groHMM also performed well at 78%. These results show that all transcription unit prediction tools had a reasonably high sensitivity for intergenic transcription, with the exception of the decision tree-based and neural network approaches (Fig 8C).

As an overall measure of transcription unit recovery, we examined the F1 score for each method, which balances precision and recall and therefore rewards methods that recover true transcription units while avoiding false positive predictions. By this metric, CGAP-HMM performed best, with groHMM and the consensus approach also performing well, whereas T-units and the random forest method showed more modest performance and the remaining machine learning approaches performed poorly (Fig 8D). To assess boundary accuracy, we next examined cumulative distribution functions of the absolute boundary errors at the TSS and PAS, where left-shifted curves indicate more accurate boundary placement because a larger fraction of predictions fall at smaller errors. At the TSS, the methods showed broadly comparable performance, although CGAP-HMM, the consensus approach, and cGAN generally placed a larger fraction of start sites closer to the annotated boundary than groHMM (Fig 8E). At the PAS, CGAP-HMM showed the strongest performance, with its curve shifted furthest to the left, indicating more accurate recovery of transcript ends, while the remaining methods showed larger end-position errors overall (Fig 8F).

Collectively, although all transcription unit identification tools performed reasonably well (aside from the decision tree-based and neural network approaches), CGAP-HMM appears to make improvements, which are in some cases substantial, on stable protein-coding genes at the expense of calling fewer very short, unstable transcription units.

The Merged, Disassociated, TUA, and % GRO-cap metrics were also used to evaluate the performance of various HMM architectures (S3A-S3C Fig), ultimately leading us to select the HMM described in Fig 3A; i.e., three states, (non-transcribed, transcribed, after element transcription), with a single covariate, (the gene starts predicted by CGAP), used to modify the transition probability from non-transcribed to transcribed states. The HMMs described in S3 Fig are by no means an exhaustive list of the architectures we experimented with, but rather serve as examples of some of the architectures we considered. As a general rule, we found the simpler models outperformed the more complex models - while it is true that our three-state model with a single covariate did better than a two-state model, increasing the number of states beyond three and the covariates beyond one resulted in poorer performance.

### 2.7 Assessing our model on other cell types/species

**Fig 9:**
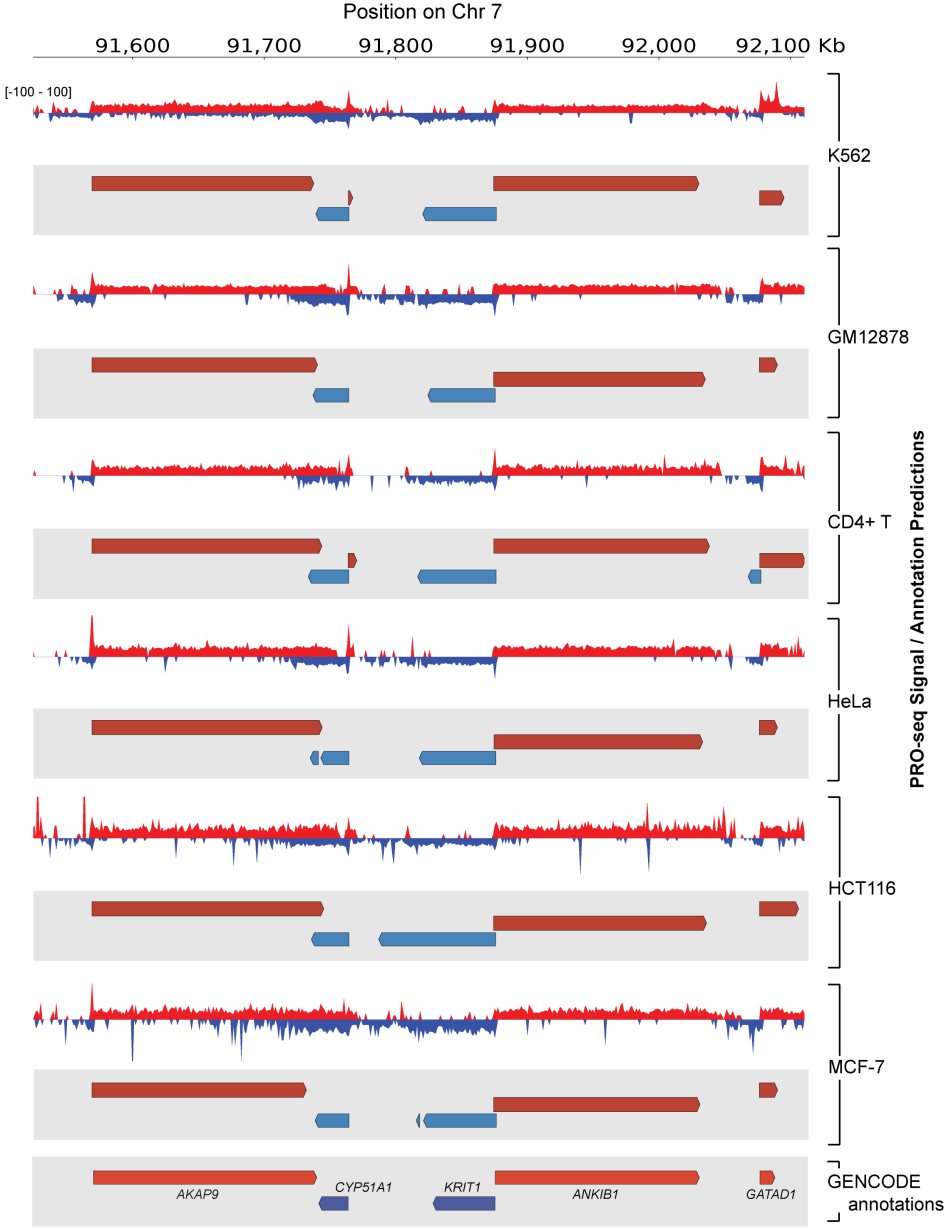
Comparison of CNN/HMM output from PRO-seq data acquired from a range of different cell types. To evaluate the overall performance of the CNN/HMM, we tested it with PRO-seq data from 5 additional cell types. Shown here are PRO-seq signals from each cell type aligned with their associated predictions for the ANKIB1 locus on chromosome 7.

**Fig 10:**
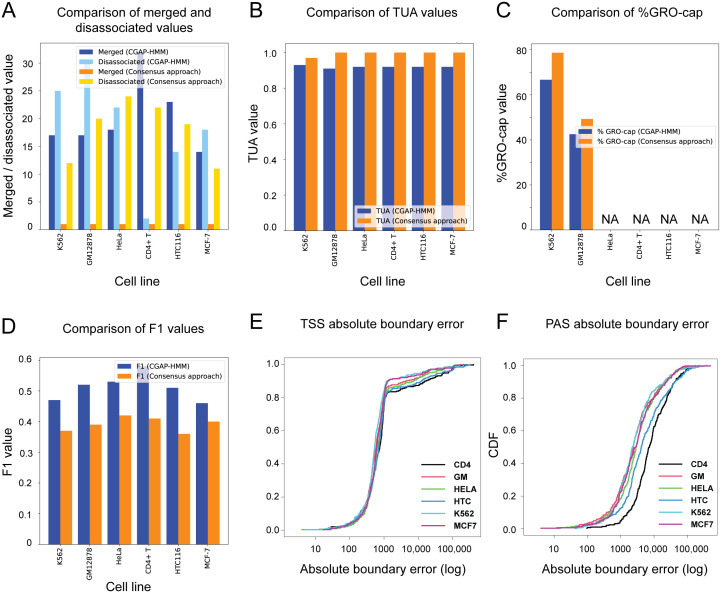
Metrics used to evaluate the performance of CGAP-HMM vs. the consensus approach for each cell type. **(A)** Comparison of merged and disassociated values. **(B)** Comparison of TUA values. **(C)** Comparison of %GRO-cap values. **(D)** F1 scores, measuring the balance between precision and sensitivity. **(E)** Cumulative distribution frequency of absolute TSS boundary errors. **(F)** Cumulative distribution frequency of absolute PAS boundary errors.

Next, we asked whether CGAP-HMM can identify the features we have discovered in the PRO-seq signal of K562 cells in other cell types/ species. To this end, we ran both our pre-trained CGAP-HMM and ensemble models on five additional cell lines: GM12878, MCF-7, HeLa, HTC116, and CD4 + T-cells (Fig 9A). To compare the results of our analysis between these cell types we ran the same evaluation metrics as used for K562 cells (Fig 10A-10F). These comparisons revealed that that method performed similarly on all cell types tested, with respect to the TUA scores. Even for a cell line that is sequenced at a much lower depth, namely the CD4 cell line, we could detect the same features using CGAP-HMM. However, values for disassociated annotations were consistently higher. We note that data for GRO-cap sites are only available for K562 and GM12878 cells, so we were unable to make the % GRO-cap recovered measurement for the remaining cell types.

**Fig 11:**
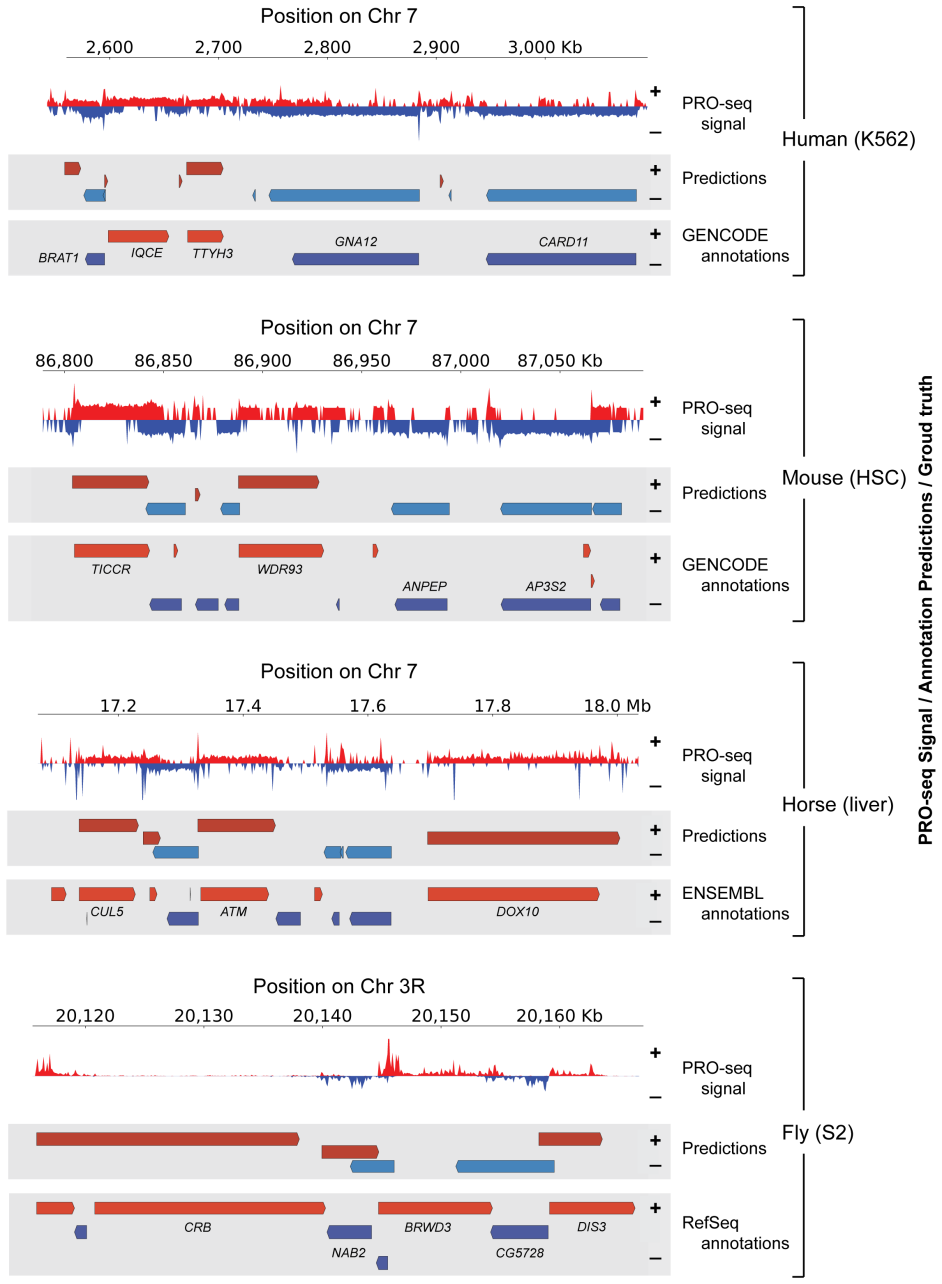
Comparison of CNN/HMM output from PRO-seq data acquired from different species. To evaluate the overall performance of the CNN/HMM, we tested it with PRO-seq data from mouse, horse, and fly. Shown here are PRO-seq signals from each cell type aligned at different loci within their respective genomes.

**Fig 12:**
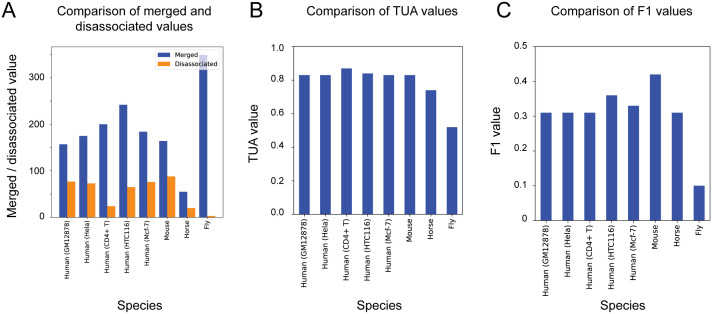
Metrics used to evaluate the performance of CGAP-HMM for each species. **(A)** Comparison of merged and disassociated values. **(B)** Comparison of TUA values. **(C)** F1 scores, measuring the balance between precision and sensitivity.

For the cross-species comparisons, we ran our pre-trained CGAP-HMM and ensemble models on mouse, horse, and fly data (Fig 11A) and evaluated the resulting transcription unit predictions using the same metrics applied to the human datasets, including merged and disassociated annotations, transcription unit accuracy, and F1 score (Fig 12A-12C). Importantly, for these species-level comparisons, the human reference set used for comparison was not filtered to retain only transcription units known to be active in K562 cells. This was done deliberately so that the human results would be directly comparable to the mouse, horse, and fly analyses, whose reference annotations were likewise not filtered for activity in the corresponding tissues or cell types. Using an unfiltered annotation framework in all species avoids introducing a bias in favor of the human dataset and ensures that differences in performance more accurately reflect the ability of the model to generalize across genomes and annotation landscapes rather than differences in reference set curation. Under this comparison scheme, CGAP-HMM performed well on mouse data, showing values for TUA and F1 score that were broadly similar to those observed in human, although with substantially higher numbers of merged and disassociated annotations. Performance on horse was weaker overall, particularly in terms of TUA, but the merged and disassociated annotation metrics were noticeably improved relative to mouse. In contrast, performance on fly was markedly worse across nearly all metrics, indicating that model transfer to Drosophila was substantially less successful than transfer to mammalian genomes.

## 3 Discussion

Gene annotators have been built by applying HMMs directly to PRO-seq data without the intermediate step of feature detection/ pattern recognition provided by a neural network. We show that by combining the feature detection strengths of neural networks with HMMs we can get improved annotations.

We evaluated two novel approaches, first a CNN coupled with an HMM. In this pipeline, the CNN and HMM serve distinct tasks. The CNN, called CGAP, was chosen as a machine learning technique that performs well for image recognition across many domains, leading us to hypothesize that it would work well in learning and classifying shapes in PRO-seq data. Indeed, we demonstrate here this approach performs well, and even with some uncertainty in the training labels can detect subtle patterns in RNA pol II distribution from the noisy PRO-seq data that allows it to classify PRO-seq into interpretable labels. The role of the HMM was intended to smooth the raw CNN output into discrete transcription unit annotations. Together, the pair performed better than existing methods which use the raw PRO-seq data as input.

We also developed a cGAN as an unsupervised alternative to the label-guided CNN + HMM pipeline. The biological motivation was to ask whether a model could discover meaningful transcriptional states directly from PRO-seq signal without manual annotation, eliminating the need for hand-curated labels and the HMM smoothing step entirely. The technical inspiration came from image segmentation, where cGANs have successfully learned to partition noisy scenes into meaningful object categories; we hypothesized that an analogous approach might recover biologically interpretable states from noisy PRO-seq data without supervision. If successful, this would represent a more generalizable solution whose learned representations could potentially be transferred to other applications. The cGAN partially succeeded in identifying transcription unit start sites but struggled to detect endpoints, breaking genes into multiple small fragments, and consequently ranked below the other methods in our benchmarks. As a result, the label-guided CNN + HMM pipeline remains the more robust solution. At the same time, the partial successes suggest that unsupervised deep learning approaches to transcription unit annotation remain worth pursuing as these methods mature.

We note that alternative architectures merit consideration. Though a simple neural network did not perform well in our initial testing, we think there are many additional architectures, such as a long short-term memory (LSTM) network, could improve its performance in this task. Replacing the HMM with an LSTM trained jointly with the CNN would yield a single end-to-end model, which is generally preferable from a machine learning standpoint as it allows the two components to be optimized together rather than sequentially. A natural next step would be to initialize such a model from the pretrained CGAP CNN via transfer learning, then fine-tune the CNN and LSTM jointly to predict transcription unit boundaries. To mitigate the ground truth labeling challenges inherent to this task, training could incorporate data augmentation strategies such as input jittering and reverse-complementing, alongside a low-parameter LSTM to reduce overfitting. We leave this as a direction for future work.

Analysis of CGAP-HMM accuracy in different species suggests that the model generalizes well across mammals without retraining the CNN, consistent with the broadly conserved genomic organization and PRO-seq signal structure within this clade. Performance is reduced in Drosophila, where the model tends to merge multiple genes into single transcription unit calls, a failure mode we attribute to the CNN having been trained on human data, in which genes are substantially larger and more spread out than in the fly genome and for which certain features like divergent transcription at promoters is missing. Extending CGAP-HMM to species with compact, gene-dense genomes will therefore require training new models on appropriate data. We propose that a natural next step is to train a single CNN on a mixture of Drosophila, *S. pombe*, and *S. cerevisiae* data: all three have tightly packaged genomes, PRO-cap or GRO-cap datasets are available for each [15,28], and together they represent the full diversity of promoter proximal pause types observed across eukaryotes [29]. We hypothesize that such a model would generalize broadly across compact eukaryotic genomes, and that together with the existing mammalian model, these two models would cover many annotation use cases across the tree of life.

Several patterns in PRO-seq data learned by CGAP are of potential interest for future study. We hypothesize that CGAP’s ability to distinguish stable from unstable transcription is driven primarily by two features: the distance transcription proceeds downstream of the TSS and the local abundance of elongating Pol II. The basis for CGAP’s ability to identify polyadenylation cleavage sites is also worth examining. This signal may reflect the brief spike in PRO-seq signal that precedes a sudden drop near the poly(A) site, which is prominent at some genes (e.g., NUPL2 in Fig 1C). PRO-seq data are inherently noisy, making both signals challenging to identify with precision; nevertheless, they likely reflect distinct modes of RNA polymerase behavior at key transcriptional transitions, and systematic study of these patterns may yield insight into the underlying transcriptional mechanics.

Overall, our data indicate that the CNN coupled with an HMM has great potential. For this approach the simple addition of more training data with better labels would improve performance. It should also be noted that the T-units method used data from more than one assay (PRO-seq and dREG), so we surmise that supplying our HMM with data from additional assays (rather than relying on the CNN’s predictions for start and end sites) may improve its performance. Our original intent was to build a method that could work in organisms where assay data is limited, but if this data is available then we may be able to improve annotations by including it. In the case of the cGAN, many of the problems we encountered in training are still active areas of research, so this approach may depend on future developments in GAN technology before it becomes a viable method for transcription unit annotation.

## 4 Methods

### 4.1 Label acquisition and processing

Training labels for the CNN and cGAN were obtained from the same datasets and then processed in different ways. We begin by outlining the initial processing steps that are common to the two methods and then discuss the differences separately.

K562 PRO-seq and GRO-seq datasets G3, G5, G6, and G7 were used for training, with GRO-seq dataset G2 used for validation, and PRO-seq dataset G1 used as a holdout dataset (see Table 1). Possible overfitting to lab specific technical variation was reduced by selecting each dataset from separate labs. Analysis done on the same datasets in [11] showed that RPKM normalized read counts were highly correlated.

We began by selecting different regions that could serve as informative training loci. Specifically, we chose regions with high PRO-seq signal, defined by previous studies as windows with more than 3 PRO-seq reads within 100 bp on a single strand or at least one read within 1000 bp on both positive and negative strands [11]. These criteria exclude regions such as the centromeric region that show little to no PRO-seq signal and thus provide little value as training labels. We expanded each of these informative regions by 20 Kb at either end into the low signal regions, so that the negative training labels (i.e., regions labeled as non-transcribed) would partially include low coverage PRO-seq signal.

We then split the informative positions into two sets - one for the plus strand and one for the minus strand. To choose training labels from these two sets we began by constructing a set of high confidence GRO-cap sites - these are GRO-cap sites that intersect with both DNase UW, and DNase Duke peak calls provided by ENCODE (see Table 1). These high-confidence GRO-cap sites were further divided into stable and unstable groups, using definitions provided in previous publications [30].

To define gene bodies, we used GENCODE annotations that overlap the region between high confidence GRO-cap sites and polyadenylation sites, and that also fall within an informative region. This subset of high confidence GENCODE annotations constitutes the gene body labels.

We constructed two distinct label types representing *cis*-regulatory elements: TSSs and transcription unit start sites. TSSs mark chromatin-accessible regions containing clusters of transcription initiation events as defined by GRO-cap; they are not strand-specific and are conceptually similar to epigenomic annotations of active regulatory elements. Transcription unit start sites, by contrast, are strand-specific and represent the initiation site of individual genes or eRNAs. These labels were derived by intersecting high-confidence GRO-cap sites with a 1 kb window centered on the 5’ end of each gene body label defined in the previous step.

For transcription end labels, we intersected polyadenylation sites (see Table 1) with a 1 kb window centered on the 3’ end of each gene body label. Active polyadenylation sites in K562 cells were identified by direct RNA sequencing [31], and tag counts were clustered and stored in the Expression and Polyadenylation Database (xPAD) [32]. Raw K562 files were downloaded from xPAD and used without further filtering (Supplementary File 1). As with transcription unit start labels, the final end labels were assigned as single-nucleotide positions marking the annotated 3’ boundary of each GENCODE transcript. After-transcription-unit labels were defined by extending a 10 kb window downstream of each end label.

To construct a set of labels for non-transcribed regions we subtracted all the labels for gene bodies, start sites, end sites, and after transcription unit regions from the genome reference. We used the set of high confidence GRO-cap sites as the set of TSS labels, and this label was excluded from non-transcribed regions on both strands.

To further filter the sets of labels, we removed from the informative positions defined at the outset any place where we have an active GENCODE annotation and no T-units prediction, and any place where we have a T-units prediction, but no active GENCODE annotation. The rational for this is T-units attempts to predict positions that are transcribed, so loci with a T-unit call (but no active GENCODE annotation) are enriched for unannotated lincRNAs and would likely confuse discriminative model training.

Finally, we compiled a list of places where each of the transcription unit bodies, transcription unit starts, transcription unit ends, after transcription unit positions, TSSs, and non-transcribed regions intersect with the filtered informative positions dataset. We associated the appropriate label with each of these intersecting regions and calculated the coverage for that region. When training, we select from these labels at random, assigning higher probability of selection to those regions with higher coverage.

When selecting the proportions of training labels to train on, we opted not to keep the exact distribution of labels within the genome. This is because the non-transcribed labels dominate the genome. We increased the representation of training labels from transcribed regions. The following proportions were used: non-transcribed training samples: 40%; transcription unit body samples: 30%; transcription unit start site samples: 5%; transcription unit end site samples: 5%; after transcription unit region samples: 5%; TSS samples: 5%; transcription unit stable start site samples: 5%; transcription unit unstable start site samples: 5%.

### 4.2 cGAN training labels

Training samples for the GAN were approached somewhat differently. Rather than giving an entire region a single label (i.e., the label associated with the data at the center of the region) as we did above, we divided the training region into 50 bp bins and assigned labels of gene body, after-gene region, and transcription start sites to each bin in the training region. Labels were assigned based on both GENCODE annotations and consensus annotations (as defined in section 4.9 *Building a consensus for CGAP-HMM, groHMM, and T-units* below). Consensus annotations performed better and are reported in section 4.10 *Accuracy metrics for transcription unit identification* below.

By way of example, the distinction between the labeling approaches can be thought of in terms of image processing of a street scene. For the CNN, the entire scene might be given a label such as “red car,” which is found at the center of the image, whereas for the GAN the training is treated as more of an image segmentation task where each object in the scene (cars, people, street signs, road and sidewalk, buildings, etc.) is given a label along with its location. In other words, labels are generated in a similar fashion to those for the CNN but presented to the GAN as a vector of values.

### 4.3 Transcription unit inference using a CNN and an HMM

We approached the problem of transcription unit inference as a two-step process. First, we predicted the locations of features such as transcription starts, transcription ends, gene body transcription, and after gene transcription based on patterns evident in the PRO-seq signal. It should be noted here that these features are subject to much of the same transcriptional noise as the original PRO-seq signal, and so cannot be used directly to infer transcriptional elements. Instead, we employ a second step where we use these features as emissions in a hidden Markov model, effectively smoothing over random spikes in the signal and/or short regions of low or zero signal. After smoothing, we infer the beginning and end of transcriptional elements as continuous regions of chromatin with well-defined start and end points.

### 4.4 CNN architecture and implementation

Architecture: The CNN consists of an initial input layer with dimensions of 1024 by 2. Prior to using the PRO-seq data as input, we bin the reads into 50 bp regions. Thus, the 1024 width of the input layer corresponds to 1024 x 50 bp, or 51.2 Kb. The height of 2 for the input corresponds to the signal on the plus and minus strands.

Following the input layer are five convolutional blocks followed by a fully connected classification stage. Each convolutional block consists of convolutional layers with rectified linear unit (ReLU) activation, batch normalization, and a 0.2x dropout. These blocks are structured as residual blocks, in which the input to each block is combined with its output through a skip connection. These residual connections facilitate the training of deeper networks by improving gradient flow and preserving informative low-level features across layers.

To increase the genomic context available to the model, the convolutions within successive residual blocks use exponentially increasing dilation rates, allowing the receptive field to expand rapidly across layers. The dilation rates were set to 1, 2, 4, 8, and 16 across the five blocks. This design enables the network to capture both short-range and long-range patterns in the PRO-seq signal while maintaining a relatively compact parameterization.

Each convolutional block is followed by a MaxPool layer that subsamples the width dimension by a factor of 2. The output of the final convolutional block is flattened and passed to a fully connected layer of 256 ReLU nodes (also with 0.2x dropout). The final output layer consists of 15 logistic regression nodes that produce the predicted probabilities for each of the 15 labels.

Training: Labeled regions were selected from a pool of approximately 740,000 labels in batches of 128. Although selection is at random, higher probability is given to regions of the PRO-seq signal with higher coverage. Once a training region has been selected, a random point within that region is chosen as the center of point of a 51.2 Kb window, that is then used as input to the CNN. It should be noted that in the case of a small label, such as a TSS of 140 bp in width, it is narrow enough that it will remain at the approximate center of the window. However, in the case of a much wider label, such as a 200 Kb gene body, the center point of the window could effectively be anywhere within the gene.

We experimented with hyper-parameter settings (convolution filter width, number of convolution layers, dropout rate, etc.) and tested these models on the validation set. We found that an input layer with 1024 nodes produced the best results, but found no other performance differences, so long as the network was sufficiently large.

The network was trained using the Adam optimizer, an adaptive learning rate optimization algorithm, specifically designed for training deep neural networks [33]. Training ran for 4,800 epochs which took approximately 5 days on an NVIDIA Tesla TITAN X (Pascal) GPU.

Evaluation: The “roc_curve” and “precision_recall_curve” functions from the scikit-learn metrics Python library [25] were used to evaluate the performance of each version of the CNN. While these functions do not give an absolute measure of how well a particular CNN is able to predict transcription units (due to uncertainty in assigning training labels), they do provide a metric for comparing relative performance of one CNN against another and thus enable us to select the “best” one. For both functions, we passed two vectors as parameters: a vector indicating the training label for the region being evaluated, and a vector of the predicted probabilities produced by the CNN for each possible label. For the roc_curve function we plotted the fraction of true positives vs. the fraction of false positives, for each of the 15 labels, at various thresholds for the label probability. We then calculated the area under the receiver operating characteristic curve (AUC) as the performance metric (larger values indicate a higher proportion of true positives vs. false positives). For the precision_recall_curve function we plotted the precision of our classifier vs. its recall (high precision indicates a low false positive rate and high recall indicates a low false negative rate). Again, we calculated the AUC and use this as a metric to assess performance (larger values indicate larger numbers of accurate results).

### 4.5 Implementation of TSS peak calling and assessment of stable and unstable transcription at cis-regulatory elements

To identify discrete transcription initiation events from the continuous CNN predictions, we performed peak calling on the CNN-derived bigWig tracks for TSS, plus-stable, minus-stable, plus-unstable, and minus-unstable transcription. For each track, the bigWig file was first converted to bedGraph format so that each 50 bp genomic bin retained its associated continuous prediction score. To reduce the contribution of low-level background signal, we estimated a score threshold from the empirical distribution of CNN predictions overlapping a high-confidence validation set of DNase I hypersensitive sites intersected with GRO-cap peaks. Bins below the selected threshold were removed, and the remaining signal was supplied to MACS3 [34] for peak calling. Peak calling was performed directly on the continuous score tracks using MACS3 with fixed-width settings appropriate for narrow transcription initiation events. Peak summits were then extracted from the MACS3 output and converted to sorted BED files, with the score assigned to each summit taken from the underlying CNN signal at the summit position.

To assess whether predicted TSS peaks were associated with stable or unstable transcription, we next compared the TSS summit set to the strand-specific stable and unstable summit sets. TSS peaks with a nearby overlapping summit in either the plus-stable or minus-stable peak set were classified as TSS-stable. We then identified TSS peaks with a nearby overlapping summit in either the plus-unstable or minus-unstable peak set and excluded any peak already assigned to the stable group; these remaining peaks were classified as TSS-unstable. This procedure allowed us to partition the non-strand-specific TSS peak calls into subsets supported by nearby stable or unstable transcription start predictions while avoiding double assignment of the same TSS peak to both groups.

To examine the chromatin context of these predicted cis-regulatory elements, we generated enrichment profile plots centered on the TSS-stable and TSS-unstable summit sets using histone modification datasets for H3K4me1, H3K4me3, and H3K27ac. Signal was summarized in windows centered on each summit and plotted as enrichment profile plots so that the distributions of promoter-associated and enhancer-associated chromatin marks could be compared between the two classes of predicted TSS peaks. These enrichment profile plots were used to assess whether CNN-defined stable and unstable transcription initiation events were associated with the expected chromatin environments.

Finally, to determine whether the original nascent transcription signal supported these classifications, we generated enrichment plots centered on the TSS-stable and TSS-unstable summit sets using the original PRO-seq data. PRO-seq signal from the plus and minus strands was summarized around each summit and plotted as aggregate enrichment profiles. These plots were used to evaluate whether the underlying PRO-seq patterns at the called summits were consistent with the expected transcriptional architectures of stable and unstable cis-regulatory elements.

### 4.6 Implementation of HMM

The primary goal of the hidden Markov model was to smooth CNN predictions into contiguous transcription unit annotations. RNA Pol II continues to actively elongate beyond the end of gene bodies, resulting in the CNN predicting continued transcription in this after-gene region. We took this into account by building a three-state model: the first state is non-transcribed, which transitions to the second state of transcribed with a probability learned by the model upon encountering an increase in PRO-seq signal and/or a CNN-predicted start site. From this transcribed state, the HMM transitions to a third state representing after-gene transcription, again using a learned probability, before finally returning to the non-transcribed state.

We explored alternative HMM structures. HMMs with only two states (non-transcribed and transcribed) performed well at identifying the beginnings of transcription units but overestimated their length. Introducing a third state allows the model to account for after-gene transcription and identify more accurate annotation endpoints. We also explored models incorporating up to 11 of the 15 CNN-predicted labels as emissions, combined with a five-state HMM (non-transcribed, transcription unit start, transcription unit body, transcription unit end, and after-transcription-unit), experimenting with various discrete and continuous emission distributions and different sets of covariates.

In some HMM architectures, covariates were used to signal state transitions. For each covariate, the maximum value within each 50 bp bin, clamped to the [0, 1] interval, was used as the transition probability between appropriate states.

Of the architectures explored, the relatively simple three-state model achieved the best performance for transcription unit annotation. In this model, strand-specific gene body outputs from the CNN were used as emission variables, modeled using gamma distributions, which are well suited to the positive-valued, right-skewed nature of these data. Strand-specific transcription unit start predictions were used as covariates to influence the transition probability from the non-transcribed to the transcribed state.

We further fine-tuned this three-state HMM by introducing state-specific emission floor parameters for the two transcribed states. Specifically, we used an offset (ε) applied to the gamma emission model so that, for a given state, the effective emission was evaluated on (x−ε)(clipped at the lower bound as required by the distribution). We denote these offsets as ε_B for the gene-body (transcribed) state and ε_D for the after-gene/decay state. Intuitively, ε_B and ε_D act as minimum-signal thresholds that reduce the likelihood that very low-amplitude CNN outputs (often attributable to background noise or residual signal) are interpreted as continued transcription, thereby controlling over-extension of predicted transcription units. Because the decay state is intended to model transitional signal near transcript termini, ε_D was constrained to be less than or equal to ε_B.

To select appropriate values, we performed a grid search over a range of ε_B and ε_D values centered around the empirical low-signal regime of the CNN gene-body track. For each parameter pair $\left(\varepsilon_{B},\varepsilon_{D}\right)$, we trained the HMM with expectation-maximization on the same chromosome-specific datasets and decoded strand-specific transcription unit annotations. We then evaluated each model against reference annotations using Transcription Unit Accuracy (TUA), which summarizes agreement between predicted and reference transcription units after accounting for fragmentation and merging, and F1 score, which balances precision and recall of predicted transcribed regions. The final ε_B and ε_D settings were chosen as those that maximized TUA while maintaining high F1, reflecting a trade-off between minimizing erroneous transcript merging/extension (precision) and preserving complete transcript coverage (recall).

All HMMs were implemented using the QHMM package (https://github.com/andrelmartins/QHMM) in R, and parameters were estimated using the Baum-Welch expectation maximization algorithm (Baum and Petrie, 1966). Evaluation of each HMM was performed using the metrics described in section 4.10, *Accuracy metrics for transcription unit identification.*

### 4.7 Implementation of cGAN

The cGAN we used was based heavily on a TensorFlow 2.0 [35] implementation of the pix2pix cGAN written by [19]. The TensorFlow implementation, copywritten in 2019 by “The TensorFlow Authors” is licensed for use under the Apache License, Version 2.0 (https://www.apache.org/licenses/LICENSE-2.0)

Architectures: The cGAN consists of two neural networks - a generator and a discriminator. They are described here with the modifications necessary for building a model that operates on the plus and minus strands of a PRO-seq dataset.

The generator was built as a modified U-NET configuration [36] - this is an encoder-decoder configuration with skip connections between the encoder layers and the corresponding decoder layers. The encoder is built from multiple convolution layers, each using a ReLU activation and each separated by a batch normalization layer. The decoder is built from multiple transposed convolution layers, also using a ReLU activation layer and each separated by a batch normalization layer, with dropout applied to the first three layers.

The discriminator is modeled on a PatchGAN [37] where each “patch” of the final output layer classifies a larger region of the input layer. Other than this, it has a similar configuration to the encoder above, with multiple convolutional layers, each using a ReLU activation, and each separated by a batch normalization layer.

Training: for a typical GAN, training takes place by alternately running the generator for a single epoch and then the discriminator for a single epoch, and continuing in this fashion so as to prevent one network getting too far ahead of the other [26].

Each example input consisted of a two-row matrix containing the PRO-seq signal from a region approximately 1.6 Mb wide (row one of the matrix was the plus strand and row two was the minus strand). For each region the generator generates an output. The discriminator is given this generated sample along with the corresponding PRO-seq signal as its first input. For the discriminator’s second input we use the input PRO-seq signal and the target annotation for same region the PRO-seq was taken from.

We can now calculate the losses for the generator and the discriminator. The loss for the discriminator is the accuracy with which it can distinguish the generated sample from the target annotation. The loss for the generator is based on its ability to generate a sample that the discriminator classifies as a real (target) annotation (i.e., how well it can fool the discriminator).

Training ran for approximately 1,000 epochs which took approximately 2 days on an NVIDIA Tesla TITAN X (Pascal) GPU.

Evaluation: Assessing the performance of a GAN by looking at the losses for the generator and discriminator can be misleading, since they fluctuate as training alternates between improving one and then the other network. As mentioned above, one problem with GAN training is one network getting too far ahead of the other: a symptom of this is the loss for one network gets very low - this happens when one network is dominating the other and is an indicator that the training of the combined network has run into problems. The authors of the Isola paper created an L1 loss for the generator that should go down as training progresses, but ultimately the measure of the combined networks performance are the metrics shown in section 4.10 *Accuracy metrics for transcription unit identification*.

### 4.8 RNA-seq based annotation using HISAT2 and BRAKER3

To generate a complementary set of annotations from RNA-seq data, we downloaded the FASTQ files for RNA-seq datasets from ENCODE (see Table 1). When multiple FASTQ files corresponded to the same RNA-seq dataset, these files were concatenated prior to alignment so that each dataset was represented by a single combined set of reads.

The concatenated RNA-seq reads were aligned to the hg19 human genome reference using HISAT2 (Kim et al., 2019). HISAT2 was selected because it is a splice-aware aligner designed for RNA-seq data and can therefore place reads that span exon-exon junctions. The output SAM/BAM files were converted to coordinate-sorted BAM files and indexed. These aligned BAM files provided the RNA-seq evidence used for BRAKER3 annotation and were also used to generate browser tracks for visual comparison with PRO-seq signal and CGAP-HMM predictions.

BRAKER3 is an automated eukaryotic gene annotation pipeline that uses extrinsic evidence, including RNA-seq alignments, to train and combine gene prediction models. In brief, BRAKER3 uses spliced RNA-seq alignments to infer expressed gene structures and to train statistical gene prediction tools including GeneMark-ETP and AUGUSTUS; resulting models are then used to predict protein-coding gene structures across the genome [27]. For this analysis, the HISAT2-aligned RNA-seq BAM files were supplied to BRAKER3 using the Galaxy implementation of BRAKER3. BRAKER3 output annotations were exported as GTF files and converted into genome browser compatible tracks. These tracks were then loaded alongside PRO-seq signal and CGAP-HMM predictions for manual inspection, and the predicted transcript spans were used for the RNA-seq-based annotation comparisons shown in Figs 5 and 6.

Because BRAKER3 is driven by RNA-seq evidence, its predictions reflect mature, spliced, steady-state RNA molecules. This makes BRAKER3 useful for defining expressed gene models and transcript structures, but it also means that its predictions are expected to differ from PRO-seq-based annotations that directly measure engaged RNA polymerase. In particular, RNA-seq alignments provide strong support for exon structure and processed transcript boundaries, whereas PRO-seq provides a more direct measurement of active transcription initiation and transcription past the polyadenylation cleavage site. This distinction motivated our use of BRAKER3 as a complementary RNA-seq-based comparison rather than as an equivalent measurement of nascent transcription.

### 4.9 Building a consensus for CGAP-HMM, groHMM, and T-units

To combine the strengths of various methods for transcription unit identification, we developed an ensemble approach to construct consensus transcription unit annotations using predictions from CGAP-HMM, groHMM, and T-units. Our approach works as follows:

- Remove small fragments (<101 bp) from each annotation dataset.
- Find the intersection of all annotations in each pair of datasets.
- Combine these intersections into a single dataset.
- Add back all transcription units from any method which does not intersect a transcription unit in the combined set. This will enable us to keep predictions made by only one method.

Examination of the results in a genome browser showed obvious cases where an annotation had been predicted by one method, but only partially predicted by the other two. However, these cases were excluded from the results because of the very small intersection of the partial predictions. To overcome this, we modified the final step of the process above to add back transcription units from any method that does not intersect a transcription unit in the combined set by more than 10%.

### 4.10 Accuracy metrics for transcription unit identification

Benchmarking in this section uses GENCODE v19 as the ground truth annotation set. For analyses in human cells, we filtered for actively transcribed isoforms by requiring a K562 GRO-cap signal on the same strand within 1 kb of the annotated TSS and a K562 polyadenylation cleavage site within 1 kb of the annotated transcript end. All isoforms meeting these criteria were retained in the benchmark set; overlapping transcripts were not removed, as this best approximates the biological ground truth in which multiple isoforms may be simultaneously active. For cross-species comparisons, where no GRO-cap or poly(A)-site sequencing data were available to filter isoforms, all transcripts were collapsed to their longest isoform. We note that this approach considerably underestimates annotation accuracy relative to the filtered human benchmark, as is apparent when comparing results across filtered and unfiltered human annotations; it was nonetheless necessary given the absence of functional data in other species.

Two of the metrics used in the evaluation of transcription unit boundaries require special explanation. The first of these measures the accuracy with which each method recovered the boundaries of gene annotations in gene-dense regions. Two types of error can arise in this measurement: either TU identification methods can erroneously break annotated genes into smaller predicted gene fragments, or alternatively a single transcription unit prediction can erroneously merge two or more separate gene annotations (S4A Fig). While the optimal method would get both values as low as possible, it should be noted that incorrect parameter tuning can result in one value decreasing at the expense of the other. For example, it would be easy to decrease the count of annotations disassociated by increasing the number of annotations merged (or vice versa), but this should be avoided.

We also used the Transcription Unit Accuracy (TUA) metric defined by [17] to evaluate TU annotation accuracy. The TUA metric implemented in the groHMM software package [17] quantifies TU annotation error in either over- or under-estimating the length of gene annotations. In brief, the TUA metric measures the accuracy of the 5’ and 3’ end of transcription unit predictions by dividing each prediction into three sections (upstream of the TSS, within the transcribed region, and downstream of the polyadenylation cleavage site). It then does the same for the ground truth annotations (we defined a set of GENCODE annotated genes which have a GRO-cap peak near the transcription start site and a poly-A RNA-seq peak near the polyadenylation cleavage site). The TUA method will penalize predicted annotations that begin before the TSS of the ground truth annotation, or that end before the polyadenylation cleavage site (S4B Fig). Since Pol II continues to transcribe past the polyadenylation cleavage site [15], TUA does not penalize for extending annotations past the polyadenylation cleavage site. The TUA metric ranges between 0 and 1, and values near 1 indicate higher agreement with the ground truth.

## 5 Data availability

All relevant data are public and GEO accession numbers can be found in Table 1.

Code Repository. All code was custom written in Python 2.7. The code and example data are available on the Danko Lab’s GitHub website (https://github.com/Danko-Lab/PROseq_Shapes) and permanently on Zenodo at https://doi.org/10.5281/zenodo.21627436.

## Supporting information

S1 Fig Precision/recall and receiver operating characteristic curves for CNN predictions. To assess the performance of the CNN, we employed several methods for testing the predictive capability of the CNN. For example, in (A) and (B) we determined the Precision recall curves for the CNN predictions for K562 and GM12878 respectively and in (C) and (D) we determined the ROC curves for K562 and GM12878 respectively.
(TIF)

S2 Fig Signed distribution of boundary errors for TSS and PAS. Negative values on the x-axis indicate the boundary error was upstream of the TSS or PAS; positive values on the x-axis indicate the boundary error was downstream of the TSS or PAS (A) Distribution of boundary errors for CGAP-HMM at the TSS. (B) Distribution of boundary errors for BRAKER3 at the TSS. (C) Distribution of boundary errors for CGAP-HMM at the PAS. (D) Distribution of boundary errors for BRAKER3 at the PAS.
(TIF)

S3 Fig Metrics used to evaluate the performance of various HMM architectures. (A) Comparison of merged and disassociated values. (B) Comparison of TUA values. (C) Comparison of %GRO-cap values.
(TIF)

S4 Fig Depiction of metrics used in the evaluation of transcription unit (TU) boundaries. (A) Merged/ disassociated TU boundaries. Different methods can erroneously break annotated TUs into smaller predicted fragments, or alternatively a single TU prediction can erroneously merge two or more separate TU annotations. (B) TUA metric. This metric quantifies TU annotation error in either over- or under-estimating the length of gene annotations. It measures the accuracy of the 5’ and 3’ end of transcription unit predictions by dividing each prediction into three sections (upstream of the TSS, within the transcribed region, and downstream of the polyadenylation cleavage site). The TUA metric will penalize predicted annotations that begin before the TSS of the ground truth annotation, or that end before the polyadenylation cleavage site. It does not penalize for extending annotations past the polyadenylation cleavage site. The TUA metric ranges between 0 and 1, and values near 1 indicate higher agreement with the ground truth.
(TIF)

S5 Data Polyadenylation cleavage sites in K562 cells. PolyA sites in K562 cells were obtained from the Expression and Polyadenylation Database (xPAD), generated by the John Lab using a snowball clustering method to process direct RNA-sequencing data. The BED file was downloaded directly from the authors’ website (http://johnlab.org/xpad/RawData/) in May 2013, and used in our paper without any additional processing.
(XLSX)
