# Supplementary material for: Accurate de novo transcription unit annotation from run-on and sequencing data
Source: PLoS Comput Biol. 2026 Aug 3;22(8):e1014559. doi: 10.1371/journal.pcbi.1014559 (PMC13460741; doi:10.1371/journal.pcbi.1014559)
Supplement: Supplementary file 1 [file pcbi.1014559.s007.docx]

**PLOS Computational Biology rebuttal**

Please include the following items when submitting your revised manuscript:

* A rebuttal letter that responds to each point raised by the editor and reviewer(s). You should upload this letter as a separate file labeled 'Response to Reviewers'. This file does not need to include responses to formatting updates and technical items listed in the 'Journal Requirements' section below.

* A marked-up copy of your manuscript that highlights changes made to the original version. You should upload this as a separate file labeled 'Revised Manuscript with Track Changes'.

* An unmarked version of your revised paper without tracked changes. You should upload this as a separate file labeled 'Manuscript'.

**Editor Comments:**

As you will see from the reviews below, the reviewers noted that the manuscript is generally sound and well-written. However, they identified a serious issue regarding the use case for the method. The manuscript motivates CGAP-HMM as identifying transcription units (rather than genes), and doing so in poorly-annotated genomes. However, the evaluations do not reflect these two motivations. A revised manuscript must precisely describe the use cases for PRO-seq-based annotation and benchmark the algorithm for those use cases.

**Journal Requirements:**

If the reviewer comments include a recommendation to cite specific previously published works, please review and evaluate these publications to determine whether they are relevant and should be cited. There is no requirement to cite these works unless the editor has indicated otherwise.

1) We ask that a manuscript source file is provided at Revision. Please upload your manuscript file as a .doc, .docx, .rtf or .tex. If you are providing a .tex file, please upload it under the item type u2018LaTeX Source Fileu2019 and leave your .pdf version as the item type u2018Manuscriptu2019.

2) Please provide an Author Summary. This should appear in your manuscript between the Abstract (if applicable) and the Introduction, and should be 150-200 words long. The aim should be to make your findings accessible to a wide audience that includes both scientists and non-scientists. Sample summaries can be found on our website under Submission Guidelines:

<https://journals.plos.org/ploscompbiol/s/submission-guidelines#loc-parts-of-a-submission>

Response: We have drafted an author summary.

3) We noticed that you used the phrase 'data not shown' in the manuscript. We do not allow these references, as the PLOS data access policy requires that all data be either published with the manuscript or made available in a publicly accessible database. Please amend the supplementary material to include the referenced data or remove the references.

Response: We have removed the reference to the data not shown.

4) Please upload all main figures as separate Figure files in .tif or .eps format. For more information about how to convert and format your figure files please see our guidelines:

<https://journals.plos.org/ploscompbiol/s/figures>

Response: We now provide each figure in a separate .tif file.

5) We notice that your supplementary Figures are included in the manuscript file. Please remove them and upload them with the file type 'Supporting Information'. Please ensure that each Supporting Information file has a legend listed in the manuscript after the references list.

Response: We have removed the supplementary figures and uploaded them separately as supporting information.

6) Please provide a detailed Financial Disclosure statement. This is published with the article. It must therefore be completed in full sentences and contain the exact wording you wish to be published.

1) Please clarify all sources of financial support for your study. List the grants, grant numbers, and organizations that funded your study, including funding received from your institution. Please note that suppliers of material support, including research materials, should be recognized in the Acknowledgements section rather than in the Financial Disclosure

2) State the initials, alongside each funding source, of each author to receive each grant. For example: "This work was supported by the National Institutes of Health (####### to AM; ###### to CJ) and the National Science Foundation (###### to AM)."

3) State what role the funders took in the study. If the funders had no role in your study, please state: "The funders had no role in study design, data collection and analysis, decision to publish, or preparation of the manuscript."

4) If any authors received a salary from any of your funders, please state which authors and which funders..

If you did not receive any funding for this study, please simply state: u201cThe authors received no specific funding for this work.u201d

Response: We now provide a financial disclosures statement.

7) Your current Financial Disclosure states, "The author(s) received no specific funding for this work.".

However, your funding information on the submission form indicates receiving fund from National Human Genome Research Institute .

Please indicate by return email the full and correct funding information for your study and confirm the order in which funding contributions should appear. Please be sure to indicate whether the funders played any role in the study design, data collection and analysis, decision to publish, or preparation of the manuscript.

Response: We have updated the funding disclosures statement.

8) Kindly revise your competing statement in the online submission form to align with the journal's style guidelines: 'The authors declare that there are no competing interests.'

Response: We have updated our competing interests statement.

**Reviewers' comments:**

Reviewer's Responses to Questions

**Comments to the Authors:
Please note here if the review is uploaded as an attachment.**

Reviewer #1: In this manuscript, the authors develop and benchmark tools for annotating the transcribed elements of a genome (genes, lncRNAs, active enhancers, etc) using PRO-seq data. Noting that PRO-seq signal is unique in its coverage of both stable and unstable transcription starts as well as gene bodies through the 3' end, they design methods to segment any raw PRO-seq track into a set of discrete annotation regions. The authors describe a major use-case of this algorithm to be identifying genes and active regulatory elements across species that are poorly annotated non-model organisms, where only one or a few experiments have been / can be performed.

Overall, the modeling strategies developed in the manuscript are sound and appropriate, the benchmarking is sensible and seems fair, and there is value in the tools and findings of the work. The authors also make clear how the authors' contributions in this manuscript are novel relative to prior work. My biggest hesitations regarding the work are based on how well it addresses its stated motivations and how clearly it describes its methods. Below, I outline concerns and suggestions, ordered from most to least significant.

Response: We thank Reviewer #1 for the thorough and constructive comments, which have informed several improvements to the revised manuscript.

M1. According to the introduction, the primary use case for the tools the authors develop and benchmark here is for the annotation of transcribed units from PRO-seq data in species with poorly annotated genomes. I agree that this would be one of the most valuable potential applications of the method. However, this main motivation is never directly addressed in the manuscript, which leads to the utility of the method being left as merely a hypothetical. The benchmarking section includes evaluation on a cross-cell-type transfer task, but there is no cross-species evaluation. Readers will have good reason to be concerned that this method may not easily transfer between divergent species, as PRO-seq profile shapes are influenced by factors such as gene-length distributions and the extent of promoter-proximal pausing, which differ across species. Thus, it would significantly strengthen the manuscript if an analysis was performed to show that this human-trained modeling method can transfer to another species (e.g. the Kwak et al. 2013 Drosophila data?) effectively, and if the authors were to include in the discussion their perspectives on how well the method will transfer to increasingly divergent species.

Response: We agree with this concern and have made several changes in response. We have added new analyses demonstrating that the CNN transfers effectively to additional mammalian species using PRO-seq data from mouse and horse without retraining; these results are presented in Fig. 11 and Fig. 12. We have also performed cross-species analyses using Drosophila data, as suggested. While the method retains predictive value in flies, the higher gene density of the fly genome leads to shorter genes being merged into longer transcription unit calls, consistent with the biological differences the reviewer identifies. We view the logical next step as training new CNN models on data from species with compact, gene-dense genomes, such as flies and yeast. We expect such new models to generalize broadly to organisms with similarly short, densely packed genomes. We discuss these limitations and proposed next steps in the revised Discussion.

M2. This work is specific to PRO-seq, and the introduction describes (fairly) that PRO-seq is uniquely valuable for generation of genomic annotations because it captures entire gene bodies as well as transcribed elements in the genome that are not genes, such as active enhancers. Given this, one would expect that at some point in the analysis, enhancers would be mentioned. However, as far as I can tell, both training of the CNN and benchmarking of all the methods focus on prediction of gene annotations, so the new method is not designed to annotate enhancers, and none of the results shown tell us how well these methods work for enhancers.

The problem with this is that PRO-seq is not the best data for gene annotations – RNA-seq clearly is. If scientists annotating a completely new genome had to choose only one experiment to do, they would much rather choose RNA-seq, which informs where the stable (likely productive) transcripts are, where and how often splicing happens, where polyadenylation happens, etc. Thus, the central motivation of this work depends on the idea that constructing annotations via PRO-seq data provides something RNA-seq can't – enhancer identification – yet the work is missing any findings supporting this.

This is fixable: given the motivations set up in the introduction, readers would have two questions raised but currently left unanswered by this work: 1) Why PRO-seq? 2) What about transcriptionally active enhancers? If the authors added additional benchmarking on enhancer annotation (potentially using the ENCODE cCRE set), both of these questions could be answered simultaneously. I do think the authors should also show some results on how well PRO-seq works as a data source for gene annotations, relative to RNA-seq (maybe this will look interesting for lncRNAs, or maybe PRO-seq can better choose which of multiple alternative TSSs is the ideal one to annotate?).

Response: We agree that the manuscript as written left two important questions unanswered (why PRO-seq? And what about enhancers?) and have added new analyses to address both.

First, we note that existing PRO-seq-based tools, including dREG, identify cis-regulatory elements, both promoters and enhancers, with high accuracy, and that performance is closely matched by the TSS calls produced by the new CGAP CNN reported here. A new feature that CGAP adds beyond existing tools is the ability to classify the stability of the resulting transcription unit, distinguishing stable from unstable transcription at each identified element. We demonstrate that these classifications reproduce the expected chromatin signatures: unstable TSS clusters are enriched for H3K4me1 relative to H3K4me3, while stable TSS clusters show the opposite pattern, consistent with the well-established distinction between enhancer and promoter-associated transcription (PMID: 25383968). These results are presented in Section 2.2 and shown in Fig. 2.

Second, we have added direct comparisons between PRO-seq and RNA-seq using human gene annotations as ground truth (Fig. 5 and 6). As expected, RNA-seq has clear advantages in certain respects: it captures internal splicing structure, which PRO-seq cannot inform, and it places the 3' ends of genes somewhat more accurately. However, we also find that PRO-seq outperforms RNA-seq at locating the 5' ends of genes, a finding we attribute to subtle 3' bias introduced by RNA degradation in RNA-seq libraries. Notably, this advantage holds even relative to high-quality ENCODE K562 RNA-seq data, suggesting it is common enough to be thought of as a systematic limitation of the assay rather than data quality per se. While long-read RNA-seq methods could in principle address this, they carry their own limitations, and the degradation problem is likely to be more pronounced in datasets generated from non-model organisms, precisely the setting where this tool is most needed.

M3. There are several ways that the description of the Methods is in need of more detail or clarification. Each individual ambiguity (enumerated below) is not catastrophic, but collectively, the lack of clarity aggregates to a major concern.

Response: We have clarified the Methods in our revised submission, as outlined below.

M3.1. Most significant Methods ambiguities needing clarification:
M3.1.1. Results section 3.4 implies a ground truth gene annotation that these methods are all being benchmarked against, but it is not clear if this ground truth is GENCODE annotations, GENCODE but filtered in any way (see M3.2.1), or some other source (I formed the impression that it was different from the CNN training labels described in section 2.1). Similarly, it is not clear what ground truth labels were used to train the HMM's emissions.

Response: We have added a paragraph to the beginning of the benchmarking section (now section 4.10) explicitly describing the ground truth annotation set and how it was constructed, including the GENCODE v19 filtering criteria and the approach used for cross-species comparisons. We have also added a forward reference to section 4.10 at the end of the HMM methods section to direct readers to the relevant accuracy metrics.

M3.1.2. Depending on what gene annotations were used: how did the authors collapse annotations per-gene where there is isoform diversity? This is particularly important for alternative TSSs, which likely show up as multiple GRO-cap peaks for a gene, since this would impact the HMM's covariate input.

Response: Transcript annotations were drawn from GENCODE, retaining all transcripts with a TSS supported by GRO-cap and a 3' end supported by a polyadenylation site. Where multiple overlapping isoforms met these criteria, all were retained rather than collapsed to a single representative in human performance evaluations, as we considered this closest to the biological ground truth. For alternative TSSs specifically, the CNN will produce distinct GRO-cap-supported peaks at each active promoter, providing independent TSS annotations that identify alternative promoter usage even where CGAP-HMM annotates the longer transcript. We have clarified this in the revised Methods section (4.10).

M3.1.3. From section 3.2: "The labels learned by CGAP that correspond to each of these states (i.e. gene bodies) were modelled as emissions using a gamma distributed random variable. " I did not understand how a gamma RV would be appropriate for modeling multi-class classification labels, as it is a continuous non-negative RV – could the authors bring up this modeling decision in a methods section and clarify its rationale?

Response: We agree that the original wording was unclear and could be misread as suggesting that discrete CGAP class labels were modeled with a gamma distribution. To clarify: the hidden states in CGAP-HMM are discrete transcriptional states (non-transcribed, gene body, and post-poly(A) transcription). Each state is associated with (or in HMM terminology, "emits”) the raw continuous non-negative CNN-derived track values for each 50 bp genomic bin, not discretized class assignments. These CNN-derived values were modeled using a gamma distribution, which we chose because they are continuous and non-negative and are empirically better described by a skewed positive distribution than a symmetric or discrete one. The HMM thus treats CNN outputs as continuous, noisy real-valued observations that are smoothed into the discrete hidden states specified by the model. We have revised the Methods and corresponding Results text to make this distinction explicit and to clarify the rationale for gamma emissions.

M3.2. More minor, but still important, Methods ambiguities needing clarification:
M3.2.1. In Table 1, please provide the accession IDs for the ENCODE datasets (the "ENCSR..." experiment name) and the specific GENCODE annotation (both version and type, e.g. "MANE Select" vs. "comprehensive" vs. "basic" - see the options here: <https://www.gencodegenes.org/human/>). The reason why this last point matters is that the authors mention that missing lncRNA annotations were relevant to filtering the CNN labels, and GENCODE recently wildly increased its lncRNA annotations (see the Kaur et al. 2024 bioRxiv preprint "GENCODE: massively expanding the lncRNA catalog through capture long-read RNA sequencing").

Response: We have added accession IDs for all ENCODE data (in GEO) and full GENCODE annotation details to Table 1 and the relevant Methods text. We used the comprehensive annotation set from GENCODE v19, which predates the substantial expansion of lncRNA annotations described in Kaur et al. 2024.

M3.2.2. Section 2.1, paragraph 6: "To build a set of transcription unit start labels we took the intersection of the high confidence GRO-cap sites with a 1 Kb window centered on the 5’ end of each of the gene body labels from the previous step. Likewise, for the set of transcription end labels we took the intersection of the polyadenylation sites (see Table 1) with a 1 Kb window centered on the 3’ end of each of the gene body labels. " From this description, it sounds like these two types of labels were created at places where a GRO-cap/polyA site was with 500bp of a GENCODE gene body annotation start/end. But how are all of the places labeled where the GRO-cap/polyA site is more than 500 bp away from the GENCODE annotation? Does this mean that every "transcription unit start" label is a 1kb wide window? If not, how wide were those areas (a histogram of the distribution of widths would be helpful)?

Response: We have clarified in the revised methods (section 4.1). The intent of this approach was to create labels only for regions where active transcription could be confidently asserted in K562 cells: because the GRO-cap and poly(A) site calls were derived from K562 data, their overlap with a GENCODE annotation provides evidence that the annotation is actively transcribed in that cell type. Regions where no such overlap was found were excluded from the label set, as transcriptional activity in K562 could not be confirmed. Once an overlap was identified, the start and end labels were assigned as single-nucleotide positions corresponding to the GENCODE annotation boundaries, not to the entire 1 kb window.

M3.2.3. Section 2.1, paragraph 6: "To construct a set of labels for non-transcribed regions we subtracted all the labels for gene bodies, start sites, end sites, and after transcription unit regions from the genome reference. Each of the above sets of labels was subsequently divided into two sets – one for the plus strand and one for the minus strand." I found this wording confusing: the first sentence describes something that I had assumed must be done in a strand-specific way already, but the second sentence implies that gene body, start site, end site, and after transcription unit annotations were determined strand-agnostically first, and after that, strand specificity was taken into consideration?

Response: The reviewer is correct that the original wording misrepresented the order of operations. Strand specificity was imposed at the outset: the plus and minus strand labels were separated first, and all subsequent label construction, including the subtraction of gene body, start site, end site, and post-transcription-unit regions to define non-transcribed labels, was performed separately for each strand. We have revised section 2.1 to reflect this, clarifying in paragraph 4 that the strand split precedes all downstream label assignments.

M3.2.4. How were PolyA sites called exactly? The authors point to polyA-RNA-seq, but how was that raw data was converted into a single coordinate for each gene body?

Response: PolyA sites were obtained from the Expression and Polyadenylation Database (xPAD), generated by the John Lab [PMID: 22753024] using a snowball clustering method to process direct RNA-sequencing data [PMID: 15647503]. We downloaded the K562 BED file directly from the authors' website (<http://johnlab.org/xpad/RawData/>) in May 2013, and used it without any additional processing. We note that the xPAD database is no longer publicly accessible and the raw files do not appear to be available through the Internet Archive; we have therefore included the poly(A) cluster files used in our analysis as a supplementary file in the revised submission. We have also clarified the source of the poly-A sequencing method in the revised Methods section and source data table.

M3.2.5. Wording in the methods (including section 2.1) describes both "transcription unit starts" and "TSSs" as if they are two different labels that the CNN predicted. The results also mention TIRs as a CNN label; it is unclear if this is the same as the "transcription unit starts". Are all of these labels different, and if so, how? It would be helpful if Figure 1A was more detailed so as to show what each of the labels would look like at an example locus.

Response: We have added a clarifying note to the Methods section (now section 4.1) distinguishing between TSSs, which mark regions of divergent transcription and are not strand-specific, and transcription unit starts, which are strand-specific and represent the CNN's strand-resolved prediction of where a transcription unit begins. These labels frequently co-occur but are not identical. We have also removed references to TIRs throughout, as the dual use of terminology was a source of unnecessary confusion. Finally, we have updated Figure 1A to illustrate each label class at an example locus, which we hope makes the distinctions among label types visually clear.

M4. There are a few places where the text describes trends without providing quantitative evidence or describes a conclusion based which I believe is not supported.
M4.1. Narrative descriptions needing more evidence: see paragraph 3 of section 3.1, wording like "Gene bodies tended to…" and the first paragraph of section 3.4, "T-units and groHMM tend to…" and "T-units frequently…"

Response: We have replaced qualitative trend language in these passages with more precise descriptions grounded in direct observation. In paragraph 3 of section 3.1 (now section 2.1), the revised text describes the browser-level correspondence between predicted labels and PRO-seq signal in specific terms: gene body signal increases at the TSS and remains elevated until near the poly(A) cleavage site, post-poly(A) signal increases as gene body signal trails off, non-transcribed labels fall in regions of absent signal on that strand, and stable versus unstable TSS states distinguish the 5' ends of long transcription units from short unstable RNAs, respectively. We follow this up with genome-wide accuracy statistics in the next paragraph. We have made analogous revisions to the first paragraph of section 3.4 to remove unsupported trend language there as well.

M4.2. The text related to Figure 4 in particular doesn't seem to really match what the figure is showing twice. For example: "T-units and groHMM tend to underestimate the lengths of transcription units with lower levels of transcription, for instance in the case of the upstream antisense transcription unit of UMAD1." The authors likely have spent much more time staring at PRO-seq tracks than me, but I just don't see how the noisy PRO-seq track here can be interpreted as a longer transcription unit than what any of these methods guessed. Also the sentence: "T-units frequently over-estimates the length of genes, incorporating post CPS transcription into the gene estimate." I wonder if the authors meant to name a different method here than T-units?
M4.3. In general, I think the authors should be encouraged to feel more comfortable describing PRO-seq data as very noisy, and thus its discretization as an inherently subjective art form. As it is, Figure 4 is accidentally anti-convincing for the purposes of benchmarking because my takeaway from it is that none of these methods is obviously superior (except that the cGAN is bad).

Response: We agree with both points M4.2 and M4.3. The example locus in Figure 7 (previously Fig. 4, referenced by the reviewer) has been replaced with a region that more clearly illustrates the characteristic failure modes of each method, specifically the tendency of T-units to fragment annotation predictions and of groHMM to merge them, making the qualitative comparison more legible. We have also added metrics to the revised Figure 8 quantifying mean error in predicted annotation start and end positions, which shows that T-units and groHMM have larger positional errors than CGAP. The text has been revised accordingly, including correction of the misattributed method name flagged in M4.2. We also take the reviewer's broader point: PRO-seq signal is inherently noisy and its discretization involves subjective judgment, and we have revised the relevant text to acknowledge this more directly rather than overstating the clarity of the signal.

M4.4. Text in the abstract: "Finally, we also report a conditional generative adversarial network (cGAN) as a generative approach to transcription unit annotation that *shows promise* for further development." Text in the discussion: "Overall, our data indicate that both the CNN coupled with an HMM, and the cGAN *have great potential*." It seemed clear from the results that the cGAN just doesn't outperform the other methods, so I think these sentences oversell that method. The conclusions related to that model might read more coherently if the authors take a stance of "our results show that deep learning is not always the winning strategy in genomics, as simpler methods outperform state of the art deep learning models for this task" (just a suggestion).

Response: We agree that the original language overstated the cGAN's performance. We have removed 'shows promise for further development' from the abstract and toned down the language in the revised Discussion, accordingly.

M5. Finally, below are suggestions of ways that the work could be strengthened further. I do not mean to imply that the authors need to do all or most of these things as a condition of acceptance.

M5.1. Modeling.
M5.1.1. The choice of using a CNN to transform noisy PRO-seq profiles via convolutional smoothing and logic over long distances, plus an HMM to smooth further and integrate information directionally across the genomic axis, is perfectly sound. However, I agree with the authors' intuition in section 3.2 that a recurrent neural network (i.e. an LSTM built on top of one or more convolutional layers) would also likely be a good fit for this task. One issue with the CNN-HMM setup is that the whole model is not trained end-to-end, which is typically considered the ideal way to train machine learning models; if the authors replaced the HMM step with an LSTM, but trained the CNN and LSTM combo simultaneously, this might outperform their current two-step strategy. The authors could try keeping the parameter count of the LSTM low and doing further data augmentation (jittering model inputs more, reverse-complementing) to account for the ground truth labeling issue. Even if the LSTM turns out to be terrible, it is valuable to show that finding, as otherwise every reader with deep learning experience will be wondering why the authors did not try an LSTM.

Response: We agree that an end-to-end CNN+LSTM architecture is a natural direction for future development, and that the lack of joint training is a limitation of the current two-step approach. Implementing and rigorously evaluating such a model is beyond the scope of the present revision, but we have added a paragraph to the Discussion noting this as a promising avenue, including the specific considerations the reviewer raises, joint training, parameter regularization, and data augmentation via jittering and reverse complementing, so that readers with deep learning backgrounds are aware that this direction has been considered.

M5.1.2. The CNN in this manuscript would likely benefit from dilated convolutions and residual connections, rather than max pooling layers, which tend to perform worse for cases where part of the objective of the model is to perform smoothing (intuitively: taking the max is not a very stable operation, as by definition it is susceptible to outliers). The authors could borrow the architecture of the bottom/first half of ClipNet, from their lab.

Response: We have incorporated residual connections and dilated convolutions into the CNN architecture. Successive residual blocks use exponentially increasing dilation rates (1, 2, 4, 8, and 16), allowing the receptive field to expand rapidly across layers and capture both short- and long-range patterns in the PRO-seq signal. Together these changes yielded a modest but significant improvement in overall performance (average precision score: 0.7228 (before) vs 0.7337 (after), p = 0.008). The updated architecture is described in section 4.4 and illustrated in Figure 1B. All results in the manuscript have been updated to reflect the revised architecture.

M5.2. Benchmarking.
M5.2.1. The authors describe computing the auROC and auPRC by literally determining the ROC and PRC curve-points, and then computing AUC from there. I believe it is recommended to directly use the functions in sci-kit-learn for auROC ("roc_auc_score") and "average precision" score (auPRC; "average_precision_score"). See the note here, paragraph starting "References Manning 2008…": <https://scikit-learn.org/stable/modules/model_evaluation.html#precision-recall-f-measure-metrics>

Response: We did use scikit-learn's built-in functions for all performance metric calculations; the custom code was used only to plot the curves. We have clarified the Methods text to make this distinction explicit, as the original wording apparently implied otherwise.

M5.2.2. I think the choice to construct the train-val-test split across datasets makes sense. I am also curious how the performance metrics would change if the split was cross-chromosome instead.

Response: Cross-chromosome splitting is standard practice for sequence-to-function models, where each input sequence is unique and memorization is a genuine concern. The situation here is meaningfully different: the input features are PRO-seq signal values, which are sparse and noisy, and offer substantially fewer opportunities for the model to memorize training examples. In related work from our group using a comparable modeling framework, we observed no performance difference between held-out and non-held-out chromosomes, suggesting overfitting of this kind is not a major concern in this setting (PMID: 35273399). Given this, we believe a cross-dataset and cross-cell-type holdout provides an equally informative and more practically relevant test of generalization, and have retained this split.

M5.3. Analysis.
M5.3.1. In results section 3.1, it is suggested that coverage best explains the difference in evaluations conducted in the training cell type, K562, vs. in GM12878. Since coverage is 4x higher in K562, the authors could test this conjecture by sub-sampling the K562 dataset down 4-fold, re-predicting the annotations, and re-computing the PRCs.

Response: We have revised the text to present sequencing depth as a likely contributing factor rather than a stated conclusion, citing prior work from our group demonstrating a clear effect of depth on related PRO-seq-based annotations (PMID: 30573452) as supporting precedent.

M5.3.2. The fact that the CNN can differentiate stable vs. unstable transcription just by looking at PRO-seq data is surprising at first. Do the authors think that this is happening because the CNN is checking for how wide the window of non-trivial PRO-seq signal after the TSS is (since unstable RNAs are shorter)? This would be an interesting hypothesis to test: one could see how stable vs. unstable transcription discrimination accuracy changes as a function of how long the PRO-seq signal continues downstream.

Response: We agree with the reviewer's intuition and think two features likely contribute to CGAP's ability to differentiate stable from unstable transcription: the distance transcription proceeds downstream of the TSS and the overall quantity of initiation at that site. To provide independent validation of the stable/unstable classification, we have added Fig. 2, which examines histone modification distributions at TSS clusters classified by CGAP. Unstable TSS clusters are enriched for H3K4me1 relative to H3K4me3, while stable TSS clusters show the opposite pattern, consistent with prior work distinguishing stable and unstable transcription (PMID: 25383968). The specific analysis the reviewer proposes, testing discrimination accuracy as a function of downstream PRO-seq signal length, would directly dissect the CNN's decision-making and is an elegant direction for future work. We have noted this in the revised Discussion alongside our hypothesis about the features driving the classification.

M5.3.3. The work discusses how the tendency of Pol II to read-through polyA sites and continue generating PRO-seq reads further downstream requires added intricacies in the method's design. I would like to get some intuition for exactly how possible it is to differentiate between "gene body" and "just downstream of polyA site" from PRO-seq data alone. The authors could provide the average ratio of PRO-seq read coverage just upstream vs. just downstream of annotated gene body ends, to see what sort of signal-to-noise ratio they are working with here.

Response: We include here a meta plot showing the average signal surrounding a polyadenylation cleavage site (see **Fig. 1**, below). The signal increases immediately following the PAS, and then decays over a period of ~5-15 kb, likely reflecting Pol II termination (covered by others, including this nice recent preprint by Robin Dowell: <https://doi.org/10.64898/2025.12.02.691879>). The pattern, though noisy, is visible at some individual genes (e.g., *NUPL2*, in Fig. 1C), and we think it likely does reflect interesting underlying biology, and is almost certainly what CGAP is learning for this feature. We agree these are interesting features to explore in depth. We now note in the revised discussion that the molecular details underlying these features should be followed up on in detail in future work.

Fig. 1: Average PRO-seq signal surrounding active polyadenylation cleavage sites (x = 0) using the G1 dataset in K562 cells.

M5.3.4. I would like to see a distribution (histogram) for how off each of the start and end coordinates of the CNN-HHM-annotations are from the semi-ground-truth annotations, in terms of signed, not absolute, distance. For example, does the distribution of residual distances between gene end coordinate annotations center on zero, or is there a systematic offset, possibly different across different methods?

Response: We have added signed distance histograms for both CGAP-HMM and BRAKER as Supplementary Fig. 2, with unsigned CDFs presented in Fig. 5. These comparisons prove informative: CGAP-HMM shows a clear advantage over RNA-seq-based annotation at gene 5' ends, while RNA-seq retains an advantage at 3' ends. These error rates sense in conjunction with the nuances of each assay, as described in response to query M2 (above).

Reviewer #2: The manuscript by Li et al. presents the CGAP-HMM deep learning/Hidden Markov Model framework for de novo annotating transcription units from genome-wide run-on sequencing datasets. The manuscript is generally well written, and the computational methods are properly implemented, however, the usefulness of the approach is doubtful.

Response: We are grateful to the reviewer for carefully reading our manuscript and providing thoughtful and constructive comments.

Major comments
1. There is a fundamental issue with the manuscript in that it tries to annotate transcription units. But how useful is that? Usually one wants a genome annotation, i.e. gene models, not transcription units. And that goal is quite well accomplished using long-read RNA sequencing, which is also certainly an easier experiment to do than doing multiple flavors of PRO-seq/PRO-cap across many cell types. Plus, of course, methods to train genome annotation models for new species based on a genome sequence and some RNA-seq data have existed for many years and are a well worked out problem.

Response: We appreciate the reviewer raising this point, as it prompted us to more directly address the relationship between transcription unit annotation and gene annotation, and to benchmark more explicitly against RNA-seq-based methods. We have revised the introduction and discussion to give a fuller account of RNA-seq as the standard approach for genome annotation, while also articulating where PRO-seq offers complementary or superior information.

To support this directly, we now benchmark CGAP-HMM against ENCODE K562 RNA-seq data processed with BRAKER3. These comparisons show that RNA-seq has a clear advantage at 3' gene boundaries, as expected given that polyadenylation signals are well captured by stable RNA. However, CGAP-HMM outperforms RNA-seq at 5' gene boundaries, likely reflecting the susceptibility of RNA-seq to degradation-induced 3' bias, a systematic limitation that is likely to be more pronounced in non-model organism datasets where sample quality is often harder to control. We also note that even long-read RNA-seq methods face challenges at TSSs due to RNA instability at the 5' end.

Beyond gene boundaries, PRO-seq uniquely captures active enhancer transcription, which RNA-seq cannot reliably detect. We argue in the revised discussion that PRO-seq is best understood as a complement to RNA-seq rather than a replacement: for researchers working in poorly annotated species where both assay types are feasible, combining them is ideal, but where only one experiment is possible, PRO-seq provides a broader picture of the active regulatory landscape than RNA-seq alone.

2. Still, let’s for the sake of the argument accept the premise that annotating transcription units is a worthwhile endeavor. Then the authors would have to demonstrate that the method is working in new species, because the human genome is pretty well annotated already. And those species would have to be something different than mammals, because in terms of genomic organization and properties all mammals are practically the same.
This the authors do not do. Even though they have a Section 3.5 titled “Assessing our model on other cell types / organisms”. But what follows is assessment in other human cell types, not other species.

Response: We agree, and have added new cross-species analyses using PRO-seq data from mouse, horse, and Drosophila. Performance transfers well to mouse and horse without retraining, consistent with the broadly conserved genomic organization across mammals. Performance is reduced in Drosophila, which we attribute primarily to the substantially higher gene density in the fly genome relative to mammals, a difference that affects both the PRO-seq signal structure and the assumptions underlying the current model architecture. We have added a paragraph to the Discussion outlining what would be required to extend CGAP-HMM to species with denser genome organization. While retraining is beyond the scope of the present work, we note that a single model trained on one or more compact genomes, such as Drosophila or yeast, would likely generalize broadly across other species with similar genomic organization, much as the current mammalian model transfers across mouse and horse. We view the development of such a model as a natural and tractable next step.

3. This is a very serious concern, because as the authors themselves say, their method “can detect subtle patterns of RNA pol II activity” and this is why it performs as well as it does. But this is in fact a problem, because the subtle patterns of RNA Pol II activity are actually quite different in different species. For example, we see in the figures how TSS annotation likely relies heavily on the property of human TSSs that they exhibit pretty strong activity in both direction, both sense and anti-sense. But this divergent transcription pattern is not universal – many other clades of eukaryotes have much more strongly unidirectional TSSs. Which has obvious implications for how the method would work once one moves away from vertebrates.

Response: We agree with this concern and view it as one of the more important limitations of the current method. The CNN was trained on human data and likely exploits features of mammalian PRO-seq signal (including the characteristic divergent transcription at TSSs) that are not universal across eukaryotes. That said, performance in Drosophila, while reduced relative to mammals, is better than one might expect given these biological differences, suggesting that other features of the PRO-seq signal (such as the step increase in signal at the 5' boundary of transcription units) provide useful information even in the absence of strong divergent transcription. We have added a discussion of these limitations, noting that the reduced performance in flies is consistent with the reviewer's interpretation. Extending the method to clades with more unidirectional TSSs or more compact genome organization will require training new CGAP models on data from organisms with these features. We propose that a natural next step is training on a mixture of Drosophila and yeast data, which would likely yield a model that generalizes broadly across compact, gene-dense genomes. Together with the existing mammalian model, these two models would cover a large fraction of the tree of life. We view this as a narrow, well-defined, and tractable direction, and have framed it as such in the Discussion.

Minor comments
1. It is impossible to read Figured 1D and all other similar figures with these colors and this thickness of the lines. The performance is clearly not all that great for all of them, but still.

Response: We have rebuilt Fig. 1D and 1E with improved colors and line weights to enhance legibility and moved them to supplemental figures S1A and S1B.

Reviewer #3: Accurate de novo transcription unit annotation from run-on and sequencing data

In this work, Munn et al develop a convolutional neural network (CGAP), train it to identify various ‘anatomical’ features of transcription units, and then develop these into transcript region annotations using an HMM. They also develop a cGAN as a generative approach to TU annotation, and present this approach as something more like a proof of concept.

I found this paper to be conceptually interesting. The main advance is in the development of the CNN-HMM hybrid. It’s a logical extension of existing ideas (feature extraction from the former, ‘smoothing’ from the latter), and so I see it more as an incremental step forward as opposed to something revolutionary. (And to be fair to the authors, they do not make audacious claims in this regard). I do see a modest but measurable improvement over groHMM / T-units, as measured. The cGAN exploration is also interesting in its own way; the approach is creative though a little underdeveloped, and I feel like what we really learn here is why this remains a hard thing to do. That said, specific observations into the present limitations / drawbacks of this sort of approach are likely to be useful for others working in the field.

The paper strikes me as methodologically sound, though I note that I’m someone who uses deep learning methods for annotation as opposed to someone who develops them from scratch. I also found it to be well written, in terms of methodology and reasoning.

Response: We thank the reviewer for carefully reading our manuscript, and for their constructive, thoughtful comments.

Here are some comments, in no particular order:

1. Ground truth dependence. The authors want the CNN to learn the anatomy of TUs purely from the shape of PRO-seq signal. But to train a supervised model, you need ground-truth labels for what each position in the genome corresponds to. The models are trained on GENCODE and GRO-cap annotations, which I think is sensible, although these data themselves have biases.

First, I would like more information about how GENCODE was used. The annotation itself is a moving target, and contains lots of different annotation ‘biotypes’ as well as models with varying levels of support. Especially, while some GENCODE models are ‘complete’ others are ‘partial,’ i.e. do not include RNA start and endpoints (i.e. TSS and pA). Did they use all GENCODE annotations? This would include pseudogenes, small RNAs etc. Was there any filtering? I appreciate that the usage of GRO-seq and pA-seq adds another layer, as these data do define such genomic sites. That said, the +/- 1kb windows used with respect to GENCODE model endpoints is quite a lot broader than the 50bp window size. It would be useful to understand how this windowing affects label precision and, in turn, model accuracy at transcriptional boundaries.

Response: We have added details on the GENCODE annotation version (v19) and filtering criteria to section 4.1 and 4.4 of the revised Methods section. We used the comprehensive annotation set of GENCODE v19 and retained all transcript biotypes. We filtered these annotations based on experimental support in K562: transcripts were required to have both a GRO-cap-supported TSS and a poly(A)-site-supported 3' end in the same cell line. Transcripts lacking either functional annotation were excluded. This cell-type-specific filtering ensures that training labels reflect actively transcribed, complete transcription units rather than the full diversity of GENCODE models. Regarding the ±1 kb windowing: the windows were used only to identify which GENCODE annotations had experimental support nearby; the final labels were assigned as single-nucleotide positions at the GENCODE annotation boundaries, not as 1 kb intervals. The precision of boundary labels is therefore not substantially affected by the window size.

Also, the stated aim is de novo annotation. But because the model is supervised on existing gene models, I wonder to what extent it might be learning the biases of GENCODE. If it is doing this, then perhaps CGAP-HMM can perform very well on loci that look like GENCODE genes, but not so well on more unusual loci. This might include, for example, genes that GENCODE have missed or not properly annotated, such as lncRNAs with lower expression, or with unusual promoters. Is there a way to look into this in more detail?

Response: We agree that this is an important consideration. Because CGAP-HMM is supervised on GENCODE annotations, it could in principle inherit biases toward well-characterized loci and perform less well on unusual or poorly annotated genes. To investigate this, we compared CGAP-HMM and BRAKER3 against LINCipedia v5.2, restricting to 1,009 filtered lincRNA annotations with GRO-cap support near the TSS and a poly(A) site near the annotated end. CGAP-HMM substantially outperformed BRAKER3 at this task (F1: 0.133 vs. 0.026). We note that F1 score likely underestimates true performance here, as novel CGAP-HMM annotations not present in LINCipedia are counted as false positives when they may instead represent genuine unannotated lincRNAs. Recall, which is less sensitive to this issue, was likewise substantially higher for CGAP-HMM than BRAKER3 (0.57 vs. 0.08). These results suggest that CGAP-HMM generalizes beyond the GENCODE training annotations and improves recovery of non-coding RNAs relative to RNA-seq-based methods.

2. On the use of labels. The model uses 15 labels derived from combinations of biological features, but the benefits of this granularity is not systematically demonstrated. Do all these components contribute in a meaningful way? Is it possible a less complex model with fewer components might work better? A general concern with highly complex models would be the potential to overfit to the data, and perhaps reduce generalisability to other usage scenarios. So I would ask the authors to do some additional work in picking apart feature importance.

Response: The 15 CNN labels were designed to provide the HMM with a rich set of candidate features spanning distinct biological states, given that PRO-seq signal is inherently noisy and a direct single-step prediction would be unreliable. Critically, we tested HMM architectures that used different combinations of the 15 labels. The best performing HMM used only gene body predictions (plus and minus strands) and gene start labels to modulate transition probabilities between non-transcribed and transcribed states. The remaining labels were effectively evaluated and set aside. The revised manuscript discusses alternative HMM architectures, some of which attempted to use the remaining labels, but none performed as well as the model we present in this paper. A summary of the metrics used to evaluate the alternative models is given in Supplemental figure 3.

3. Rational and presentation of the cGAN model. As noted, I found this section interesting but rather underdeveloped, especially in view of the fact that the authors ultimately find aspects of instability and underperformance. One thing I found myself missing here was an explicit statement of purpose; i.e. what exactly is the biological hypothesis being tested here? What is the precise biological motivation for adversarial training, given that there are other alternatives? Above all, why? I appreciate that there is some informative commentary to this effect, but - given the rather uncertain outcomes here - I think this section needs something of a narrative re-think. At the moment, it comes across as an exploratory add-on rather than a methodically grounded alternative.

Response: The biological motivation for the cGAN approach is as follows: rather than hand-tuning 15 labels for the CNN as described in Response 2, we asked whether a model could learn informative labels directly from PRO-seq signal without manual annotation, eliminating the need for the HMM pipeline entirely. If successful, this would represent a more elegant and generalizable solution, and the learned labels could potentially be interpreted and transferred to other applications. The technical inspiration was adversarial training frameworks from image processing, where cGANs have successfully learned to segment noisy scenes into meaningful object categories; we hypothesized that an analogous approach might allow the model to discover biologically meaningful states in noisy PRO-seq data without supervision.

As the reviewer notes, the results showed instability and underperformance relative to our HMM-based approach. We present this as an informative negative result: the noise characteristics of PRO-seq signal appear to pose challenges that current cGAN architectures do not handle well, and this comparison clarifies why the structured, label-guided HMM pipeline remains the more robust solution. We have restructured this section in the revised manuscript to make the biological question, the motivation for adversarial training, and the interpretation of the negative result explicit from the outset, so that the cGAN analysis reads as a methodologically grounded alternative hypothesis rather than an exploratory addition.

4. Additional biological validation. Their model validation relies on overlap with existing annotations (GENCODE, GRO-cap, PolyA), which are the same datasets used for training. What I miss is independent biological confirmation, the lack of which makes it hard to tell whether the model is generalising or memorising. Ways to look at this in more detail could include an examination of nascent transcription in other species, or looking at RNA-seq from unannotated samples.

In fact, I could see other ‘gains’ in testing the model on other species, i.e. applying their human-models to (e.g.) mouse and comparing against native annotations. Signal patterns in Pro-seq should be conserved, after all, and annotation in other species seems to be part of the overall goal with this work. If they don’t want to do this, some discussion of potential challenges here would be useful.

Response: We thank the reviewer for this suggestion. In response, we have applied CGAP-HMM to mouse, horse, and fly PRO-seq datasets and present these results in Figs. 11 and 12 (Results section 2.7). Performance was similar across all mammalian species, supporting generalizability of the approach beyond human. The reduced performance in fly reflects the substantially distinct gene structure in Drosophila rather than a failure of generalization, as the model was designed around mammalian transcription architecture.

5. Using the models for actual annotation. ‘Our original intent was to build a method that could work in organisms where assay data is limited’. I’d like to know more about the thinking here. Gene annotation in practice is a more complex process than their approximation with this work, which is really about gene boundaries. For example, annotation defines not just regions of transcription but also precisely defined spliced sites based on transcriptomics data. (Of course, Pro-seq is not suitable for looking at splicing). How could they envisage their methods dovetailing with existing workflows? And, as noted above, the potential impact of the method would appear stronger if the authors could explicitly show how TU predictions could seed or improve gene calling in non-model species.

Response: We agree with the reviewer's point and appreciate the opportunity to clarify. We do not envision CGAP-HMM as a standalone replacement for existing annotation workflows, but rather as a complementary tool that improves specific aspects of annotation where PRO-seq has unique advantages. In the revised manuscript, we now compare CGAP-HMM output directly with RNA-seq-based annotation pipelines: TU predictions improve promoter boundary annotation relative to BRAKER3 (Fig. 5 and 6), demonstrating concrete utility within existing workflows. We fully agree that PRO-seq is not suitable for defining splice sites, and we do not claim otherwise.

Beyond gene boundaries, PRO-seq uniquely identifies active enhancers through bidirectional unstable transcription, and CGAP-HMM extends this by distinguishing stable from unstable RNA genome-wide (Fig. 2), a capability RNA-seq pipelines lack entirely. We therefore envision the most powerful use case as a combination of PRO-seq-based CGAP-HMM annotation with RNA-seq pipelines, jointly improving both gene and epigenomic annotation in non-model organisms where comprehensive assay data are limited. We now discuss this explicitly in the revised discussion.
