# Supplementary material for: Accurate de novo transcription unit annotation from run-on and sequencing data
Source: PLoS Comput Biol. 2026 Aug 3;22(8):e1014559. doi: 10.1371/journal.pcbi.1014559 (PMC13460741; doi:10.1371/journal.pcbi.1014559)
Supplement: Peer review history

# Peer review: Decision Letter 0

**DOI:** 10.1371/journal.pcbi.1014559.r001

**Authors:** Shaun Mahony, Maxwell Libbrecht

3 Nov 2025

Accurate de novo transcription unit annotation from run-on and sequencing data

PLOS Computational Biology

Dear Dr. Danko,

Thank you for submitting your manuscript to PLOS Computational Biology. After careful consideration, we feel that it has merit but does not fully meet PLOS Computational Biology's publication criteria as it currently stands. Therefore, we invite you to submit a revised version of the manuscript that addresses the points raised during the review process.

Please submit your revised manuscript within 60 days Jan 03 2026 11:59PM. If you will need more time than this to complete your revisions, please reply to this message or contact the journal office at ploscompbiol@plos.org. When you're ready to submit your revision, log on to https://www.editorialmanager.com/pcompbiol/ and select the 'Submissions Needing Revision' folder to locate your manuscript file.

Please include the following items when submitting your revised manuscript:

* A rebuttal letter that responds to each point raised by the editor and reviewer(s). You should upload this letter as a separate file labeled 'Response to Reviewers'. This file does not need to include responses to formatting updates and technical items listed in the 'Journal Requirements' section below.

* A marked-up copy of your manuscript that highlights changes made to the original version. You should upload this as a separate file labeled 'Revised Manuscript with Track Changes'.

* An unmarked version of your revised paper without tracked changes. You should upload this as a separate file labeled 'Manuscript'.

If you would like to make changes to your financial disclosure, competing interests statement, or data availability statement, please make these updates within the submission form at the time of resubmission. Guidelines for resubmitting your figure files are available below the reviewer comments at the end of this letter

We look forward to receiving your revised manuscript.

Kind regards,

Maxwell Wing Libbrecht, Ph.D.

Academic Editor

PLOS Computational Biology

Shaun Mahony

Section Editor

PLOS Computational Biology

**Additional Editor Comments:**

As you will see from the reviews below, the reviewers noted that the manuscript is generally sound and well-written. However, they identified a serious issue regarding the use case for the method. The manuscript motivates CGAP-HMM as identifying transcription units (rather than genes), and doing so in poorly-annotated genomes. However, the evaluations do not reflect these two motivations. A revised manuscript must precisely describe the use cases for PRO-seq-based annotation and benchmark the algorithm for those use cases.

**Journal Requirements:**

**Reviewers' comments:**

Reviewer's Responses to Questions

**Comments to the Authors:**

**Please note here if the review is uploaded as an attachment.**

Reviewer #1: In this manuscript, the authors develop and benchmark tools for annotating the transcribed elements of a genome (genes, lncRNAs, active enhancers, etc) using PRO-seq data. Noting that PRO-seq signal is unique in its coverage of both stable and unstable transcription starts as well as gene bodies through the 3' end, they design methods to segment any raw PRO-seq track into a set of discrete annotation regions. The authors describe a major use-case of this algorithm to be identifying genes and active regulatory elements across species that are poorly annotated non-model organisms, where only one or a few experiments have been / can be performed.

Overall, the modeling strategies developed in the manuscript are sound and appropriate, the benchmarking is sensible and seems fair, and there is value in the tools and findings of the work. The authors also make clear how the authors' contributions in this manuscript are novel relative to prior work. My biggest hesitations regarding the work are based on how well it addresses its stated motivations and how clearly it describes its methods. Below, I outline concerns and suggestions, ordered from most to least significant.

M1. According to the introduction, the primary use case for the tools the authors develop and benchmark here is for the annotation of transcribed units from PRO-seq data in species with poorly annotated genomes. I agree that this would be one of the most valuable potential applications of the method. However, this main motivation is never directly addressed in the manuscript, which leads to the utility of the method being left as merely a hypothetical. The benchmarking section includes evaluation on a cross-cell-type transfer task, but there is no cross-species evaluation. Readers will have good reason to be concerned that this method may not easily transfer between divergent species, as PRO-seq profile shapes are influenced by factors such as gene-length distributions and the extent of promoter-proximal pausing, which differ across species. Thus, it would significantly strengthen the manuscript if an analysis was performed to show that this human-trained modeling method can transfer to another species (e.g. the Kwak et al. 2013 Drosophila data?) effectively, and if the authors were to include in the discussion their perspectives on how well the method will transfer to increasingly divergent species.

M2. This work is specific to PRO-seq, and the introduction describes (fairly) that PRO-seq is uniquely valuable for generation of genomic annotations because it captures entire gene bodies as well as transcribed elements in the genome that are not genes, such as active enhancers. Given this, one would expect that at some point in the analysis, enhancers would be mentioned. However, as far as I can tell, both training of the CNN and benchmarking of all the methods focus on prediction of gene annotations, so the new method is not designed to annotate enhancers, and none of the results shown tell us how well these methods work for enhancers.

The problem with this is that PRO-seq is not the best data for gene annotations – RNA-seq clearly is. If scientists annotating a completely new genome had to choose only one experiment to do, they would much rather choose RNA-seq, which informs where the stable (likely productive) transcripts are, where and how often splicing happens, where polyadenylation happens, etc. Thus, the central motivation of this work depends on the idea that constructing annotations via PRO-seq data provides something RNA-seq can't – enhancer identification – yet the work is missing any findings supporting this.

This is fixable: given the motivations set up in the introduction, readers would have two questions raised but currently left unanswered by this work: 1) Why PRO-seq? 2) What about transcriptionally active enhancers? If the authors added additional benchmarking on enhancer annotation (potentially using the ENCODE cCRE set), both of these questions could be answered simultaneously. I do think the authors should also show some results on how well PRO-seq works as a data source for gene annotations, relative to RNA-seq (maybe this will look interesting for lncRNAs, or maybe PRO-seq can better choose which of multiple alternative TSSs is the ideal one to annotate?).

M3. There are several ways that the description of the Methods is in need of more detail or clarification. Each individual ambiguity (enumerated below) is not catastrophic, but collectively, the lack of clarity aggregates to a major concern.

M3.1. Most significant Methods ambiguities needing clarification:

M3.1.1. Results section 3.4 implies a ground truth gene annotation that these methods are all being benchmarked against, but it is not clear if this ground truth is GENCODE annotations, GENCODE but filtered in any way (see M3.2.1), or some other source (I formed the impression that it was different from the CNN training labels described in section 2.1). Similarly, it is not clear what ground truth labels were used to train the HMM's emissions.

M3.1.2. Depending on what gene annotations were used: how did the authors collapse annotations per-gene where there is isoform diversity? This is particularly important for alternative TSSs, which likely show up as multiple GRO-cap peaks for a gene, since this would impact the HMM's covariate input.

M3.1.3. From section 3.2: "The labels learned by CGAP that correspond to each of these states (i.e. gene bodies) were modelled as emissions using a gamma distributed random variable. " I did not understand how a gamma RV would be appropriate for modeling multi-class classification labels, as it is a continuous non-negative RV – could the authors bring up this modeling decision in a methods section and clarify its rationale?

M3.2. More minor, but still important, Methods ambiguities needing clarification:

M3.2.1. In Table 1, please provide the accession IDs for the ENCODE datasets (the "ENCSR..." experiment name) and the specific GENCODE annotation (both version and type, e.g. "MANE Select" vs. "comprehensive" vs. "basic" - see the options here: https://www.gencodegenes.org/human/). The reason why this last point matters is that the authors mention that missing lncRNA annotations were relevant to filtering the CNN labels, and GENCODE recently wildly increased its lncRNA annotations (see the Kaur et al. 2024 bioRxiv preprint "GENCODE: massively expanding the lncRNA catalog through capture long-read RNA sequencing").

M3.2.2. Section 2.1, paragraph 6: "To build a set of transcription unit start labels we took the intersection of the high confidence GRO-cap sites with a 1 Kb window centered on the 5’ end of each of the gene body labels from the previous step. Likewise, for the set of transcription end labels we took the intersection of the polyadenylation sites (see Table 1) with a 1 Kb window centered on the 3’ end of each of the gene body labels. " From this description, it sounds like these two types of labels were created at places where a GRO-cap/polyA site was with 500bp of a GENCODE gene body annotation start/end. But how are all of the places labeled where the GRO-cap/polyA site is more than 500 bp away from the GENCODE annotation? Does this mean that every "transcription unit start" label is a 1kb wide window? If not, how wide were those areas (a histogram of the distribution of widths would be helpful)?

M3.2.3. Section 2.1, paragraph 6: "To construct a set of labels for non-transcribed regions we subtracted all the labels for gene bodies, start sites, end sites, and after transcription unit regions from the genome reference. Each of the above sets of labels was subsequently divided into two sets – one for the plus strand and one for the minus strand." I found this wording confusing: the first sentence describes something that I had assumed must be done in a strand-specific way already, but the second sentence implies that gene body, start site, end site, and after transcription unit annotations were determined strand-agnostically first, and after that, strand specificity was taken into consideration?

M3.2.4. How were PolyA sites called exactly? The authors point to polyA-RNA-seq, but how was that raw data was converted into a single coordinate for each gene body?

M3.2.5. Wording in the methods (including section 2.1) describes both "transcription unit starts" and "TSSs" as if they are two different labels that the CNN predicted. The results also mention TIRs as a CNN label; it is unclear if this is the same as the "transcription unit starts". Are all of these labels different, and if so, how? It would be helpful if Figure 1A was more detailed so as to show what each of the labels would look like at an example locus.

M4. There are a few places where the text describes trends without providing quantitative evidence or describes a conclusion based which I believe is not supported.

M4.1. Narrative descriptions needing more evidence: see paragraph 3 of section 3.1, wording like "Gene bodies tended to…" and the first paragraph of section 3.4, "T-units and groHMM tend to…" and "T-units frequently…"

M4.2. The text related to Figure 4 in particular doesn't seem to really match what the figure is showing twice. For example: "T-units and groHMM tend to underestimate the lengths of transcription units with lower levels of transcription, for instance in the case of the upstream antisense transcription unit of UMAD1." The authors likely have spent much more time staring at PRO-seq tracks than me, but I just don't see how the noisy PRO-seq track here can be interpreted as a longer transcription unit than what any of these methods guessed. Also the sentence: "T-units frequently over-estimates the length of genes, incorporating post CPS transcription into the gene estimate." I wonder if the authors meant to name a different method here than T-units?

M4.3. In general, I think the authors should be encouraged to feel more comfortable describing PRO-seq data as very noisy, and thus its discretization as an inherently subjective art form. As it is, Figure 4 is accidentally anti-convincing for the purposes of benchmarking because my takeaway from it is that none of these methods is obviously superior (except that the cGAN is bad).

M4.4. Text in the abstract: "Finally, we also report a conditional generative adversarial network (cGAN) as a generative approach to transcription unit annotation that *shows promise* for further development." Text in the discussion: "Overall, our data indicate that both the CNN coupled with an HMM, and the cGAN *have great potential*." It seemed clear from the results that the cGAN just doesn't outperform the other methods, so I think these sentences oversell that method. The conclusions related to that model might read more coherently if the authors take a stance of "our results show that deep learning is not always the winning strategy in genomics, as simpler methods outperform state of the art deep learning models for this task" (just a suggestion).

M5. Finally, below are suggestions of ways that the work could be strengthened further. I do not mean to imply that the authors need to do all or most of these things as a condition of acceptance.

M5.1. Modeling.

M5.1.1. The choice of using a CNN to transform noisy PRO-seq profiles via convolutional smoothing and logic over long distances, plus an HMM to smooth further and integrate information directionally across the genomic axis, is perfectly sound. However, I agree with the authors' intuition in section 3.2 that a recurrent neural network (i.e. an LSTM built on top of one or more convolutional layers) would also likely be a good fit for this task. One issue with the CNN-HMM setup is that the whole model is not trained end-to-end, which is typically considered the ideal way to train machine learning models; if the authors replaced the HMM step with an LSTM, but trained the CNN and LSTM combo simultaneously, this might outperform their current two-step strategy. The authors could try keeping the parameter count of the LSTM low and doing further data augmentation (jittering model inputs more, reverse-complementing) to account for the ground truth labeling issue. Even if the LSTM turns out to be terrible, it is valuable to show that finding, as otherwise every reader with deep learning experience will be wondering why the authors did not try an LSTM.

M5.1.2. The CNN in this manuscript would likely benefit from dilated convolutions and residual connections, rather than max pooling layers, which tend to perform worse for cases where part of the objective of the model is to perform smoothing (intuitively: taking the max is not a very stable operation, as by definition it is susceptible to outliers). The authors could borrow the architecture of the bottom/first half of ClipNet, from their lab.

M5.2. Benchmarking.

M5.2.1. The authors describe computing the auROC and auPRC by literally determining the ROC and PRC curve-points, and then computing AUC from there. I believe it is recommended to directly use the functions in sci-kit-learn for auROC ("roc_auc_score") and "average precision" score (auPRC; "average_precision_score"). See the note here, paragraph starting "References Manning 2008…": https://scikit-learn.org/stable/modules/model_evaluation.html#precision-recall-f-measure-metrics

M5.2.2. I think the choice to construct the train-val-test split across datasets makes sense. I am also curious how the performance metrics would change if the split was cross-chromosome instead.

M5.3. Analysis.

M5.3.1. In results section 3.1, it is suggested that coverage best explains the difference in evaluations conducted in the training cell type, K562, vs. in GM12878. Since coverage is 4x higher in K562, the authors could test this conjecture by sub-sampling the K562 dataset down 4-fold, re-predicting the annotations, and re-computing the PRCs.

M5.3.2. The fact that the CNN can differentiate stable vs. unstable transcription just by looking at PRO-seq data is surprising at first. Do the authors think that this is happening because the CNN is checking for how wide the window of non-trivial PRO-seq signal after the TSS is (since unstable RNAs are shorter)? This would be an interesting hypothesis to test: one could see how stable vs. unstable transcription discrimination accuracy changes as a function of how long the PRO-seq signal continues downstream.

M5.3.3. The work discusses how the tendency of Pol II to read-through polyA sites and continue generating PRO-seq reads further downstream requires added intricacies in the method's design. I would like to get some intuition for exactly how possible it is to differentiate between "gene body" and "just downstream of polyA site" from PRO-seq data alone. The authors could provide the average ratio of PRO-seq read coverage just upstream vs. just downstream of annotated gene body ends, to see what sort of signal-to-noise ratio they are working with here.

M5.3.4. I would like to see a distribution (histogram) for how off each of the start and end coordinates of the CNN-HHM-annotations are from the semi-ground-truth annotations, in terms of signed, not absolute, distance. For example, does the distribution of residual distances between gene end coordinate annotations center on zero, or is there a systematic offset, possibly different across different methods?

Reviewer #2: The manuscript by Li et al. presents the CGAP-HMM deep learning/Hidden Markov Model framework for de novo annotating transcription units from genome-wide run-on sequencing datasets. The manuscript is generally well written, and the computational methods are properly implemented, however, the usefulness of the approach is doubtful.

Major comments

1. There is a fundamental issue with the manuscript in that it tries to annotate transcription units. But how useful is that? Usually one wants a genome annotation, i.e. gene models, not transcription units. And that goal is quite well accomplished using long-read RNA sequencing, which is also certainly an easier experiment to do than doing multiple flavors of PRO-seq/PRO-cap across many cell types. Plus, of course, methods to train genome annotation models for new species based on a genome sequence and some RNA-seq data have existed for many years and are a well worked out problem.

2. Still, let’s for the sake of the argument accept the premise that annotating transcription units is a worthwhile endeavor. Then the authors would have to demonstrate that the method is working in new species, because the human genome is pretty well annotated already. And those species would have to be something different than mammals, because in terms of genomic organization and properties all mammals are practically the same.

This the authors do not do. Even though they have a Section 3.5 titled “Assessing our model on other cell types / organisms”. But what follows is assessment in other human cell types, not other species.

3. This is a very serious concern, because as the authors themselves say, their method “can detect subtle patterns of RNA pol II activity” and this is why it performs as well as it does. But this is in fact a problem, because the subtle patterns of RNA Pol II activity are actually quite different in different species. For example, we see in the figures how TSS annotation likely relies heavily on the property of human TSSs that they exhibit pretty strong activity in both direction, both sense and anti-sense. But this divergent transcription pattern is not universal – many other clades of eukaryotes have much more strongly unidirectional TSSs. Which has obvious implications for how the method would work once one moves away from vertebrates.

Minor comments

1. It is impossible to read Figured 1D and all other similar figures with these colors and this thickness of the lines. The performance is clearly not all that great for all of them, but still.

Reviewer #3: Accurate de novo transcription unit annotation from run-on and sequencing data

In this work, Munn et al develop a convolutional neural network (CGAP), train it to identify various ‘anatomical’ features of transcription units, and then develop these into transcript region annotations using an HMM. They also develop a cGAN as a generative approach to TU annotation, and present this approach as something more like a proof of concept.

I found this paper to be conceptually interesting. The main advance is in the development of the CNN-HMM hybrid. It’s a logical extension of existing ideas (feature extraction from the former, ‘smoothing’ from the latter), and so I see it more as an incremental step forward as opposed to something revolutionary. (And to be fair to the authors, they do not make audacious claims in this regard). I do see a modest but measurable improvement over groHMM / T-units, as measured. The cGAN exploration is also interesting in its own way; the approach is creative though a little underdeveloped, and I feel like what we really learn here is why this remains a hard thing to do. That said, specific observations into the present limitations / drawbacks of this sort of approach are likely to be useful for others working in the field.

The paper strikes me as methodologically sound, though I note that I’m someone who uses deep learning methods for annotation as opposed to someone who develops them from scratch. I also found it to be well written, in terms of methodology and reasoning.

Here are some comments, in no particular order:

1. Ground truth dependence. The authors want the CNN to learn the anatomy of TUs purely from the shape of PRO-seq signal. But to train a supervised model, you need ground-truth labels for what each position in the genome corresponds to. The models are trained on GENCODE and GRO-cap annotations, which I think is sensible, although these data themselves have biases.

First, I would like more information about how GENCODE was used. The annotation itself is a moving target, and contains lots of different annotation ‘biotypes’ as well as models with varying levels of support. Especially, while some GENCODE models are ‘complete’ others are ‘partial,’ i.e. do not include RNA start and endpoints (i.e. TSS and pA). Did they use all GENCODE annotations? This would include pseudogenes, small RNAs etc. Was there any filtering? I appreciate that the usage of GRO-seq and pA-seq adds another layer, as these data do define such genomic sites. That said, the +/- 1kb windows used with respect to GENCODE model endpoints is quite a lot broader than the 50bp window size. It would be useful to understand how this windowing affects label precision and, in turn, model accuracy at transcriptional boundaries.

Also, the stated aim is de novo annotation. But because the model is supervised on existing gene models, I wonder to what extent it might be learning the biases of GENCODE. If it is doing this, then perhaps CGAP-HMM can perform very well on loci that look like GENCODE genes, but not so well on more unusual loci. This might include, for example, genes that GENCODE have missed or not properly annotated, such as lncRNAs with lower expression, or with unusual promoters. Is there a way to look into this in more detail?

2. On the use of labels. The model uses 15 labels derived from combinations of biological features, but the benefits of this granularity is not systematically demonstrated. Do all these components contribute in a meaningful way? Is it possible a less complex model with fewer components might work better? A general concern with highly complex models would be the potential to overfit to the data, and perhaps reduce generalisability to other usage scenarios. So I would ask the authors to do some additional work in picking apart feature importance.

3. Rational and presentation of the cGAN model. As noted, I found this section interesting but rather underdeveloped, especially in view of the fact that the authors ultimately find aspects of instability and underperformance. One thing I found myself missing here was an explicit statement of purpose; i.e. what exactly is the biological hypothesis being tested here? What is the precise biological motivation for adversarial training, given that there are other alternatives? Above all, why? I appreciate that there is some informative commentary to this effect, but - given the rather uncertain outcomes here - I think this section needs something of a narrative re-think. At the moment, it comes across as an exploratory add-on rather than a methodically grounded alternative.

4. Additional biological validation. Their model validation relies on overlap with existing annotations (GENCODE, GRO-cap, PolyA), which are the same datasets used for training. What I miss is independent biological confirmation, the lack of which makes it hard to tell whether the model is generalising or memorising. Ways to look at this in more detail could include an examination of nascent transcription in other species, or looking at RNA-seq from unannotated samples.

In fact, I could see other ‘gains’ in testing the model on other species, i.e. applying their human-models to (e.g.) mouse and comparing against native annotations. Signal patterns in Pro-seq should be conserved, after all, and annotation in other species seems to be part of the overall goal with this work. If they don’t want to do this, some discussion of potential challenges here would be useful.

5. Using the models for actual annotation. ‘Our original intent was to build a method that could work in organisms where assay data is limited’. I’d like to know more about the thinking here. Gene annotation in practice is a more complex process than their approximation with this work, which is really about gene boundaries. For example, annotation defines not just regions of transcription but also precisely defined spliced sites based on transcriptomics data. (Of course, Pro-seq is not suitable for looking at splicing). How could they envisage their methods dovetailing with existing workflows? And, as noted above, the potential impact of the method would appear stronger if the authors could explicitly show how TU predictions could seed or improve gene calling in non-model species.

**********

**Have the authors made all data and (if applicable) computational code underlying the findings in their manuscript fully available?**

The PLOS Data policy requires authors to make all data and code underlying the findings described in their manuscript fully available without restriction, with rare exception (please refer to the Data Availability Statement in the manuscript PDF file). The data and code should be provided as part of the manuscript or its supporting information, or deposited to a public repository. For example, in addition to summary statistics, the data points behind means, medians and variance measures should be available. If there are restrictions on publicly sharing data or code —e.g. participant privacy or use of data from a third party—those must be specified.

Reviewer #1: Yes

Reviewer #2: Yes

Reviewer #3: Yes

**********

PLOS authors have the option to publish the peer review history of their article (what does this mean?). If published, this will include your full peer review and any attached files.

If you choose “no”, your identity will remain anonymous but your review may still be made public.

**Do you want your identity to be public for this peer review?**  For information about this choice, including consent withdrawal, please see our Privacy Policy.

Reviewer #1: No

Reviewer #2: No

Reviewer #3: No

**Figure resubmission:**

After uploading your figures to PLOS’s NAAS tool - https://ngplosjournals.pagemajik.ai/artanalysis, NAAS will process the files provided and display the results in the "Uploaded Files" section of the page as the processing is complete. If the uploaded figures meet our requirements (or NAAS is able to fix the files to meet our requirements), the figure will be marked as "fixed" above. If NAAS is unable to fix the files, a red "failed" label will appear above. When NAAS has confirmed that the figure files meet our requirements, please download the file via the download option, and include these NAAS processed figure files when submitting your revised manuscript.

**Reproducibility:**

To enhance the reproducibility of your results, we recommend that authors of applicable studies deposit laboratory protocols in protocols.io, where a protocol can be assigned its own identifier (DOI) such that it can be cited independently in the future. Additionally, PLOS ONE offers an option to publish peer-reviewed clinical study protocols. Read more information on sharing protocols at https://plos.org/protocols?utm_medium=editorial-email&utm_source=authorletters&utm_campaign=protocols

# Author comment: Author response to Decision Letter 1

**DOI:** 10.1371/journal.pcbi.1014559.r002

14 May 2026

Attachment Submitted filename: Munn-Reviewer-Response.docx

# Peer review: Decision Letter 1

**DOI:** 10.1371/journal.pcbi.1014559.r003

**Authors:** Shaun Mahony

9 Jul 2026

Dear Professor Danko,

We are pleased to inform you that your manuscript 'Accurate de novo transcription unit annotation from run-on and sequencing data' has been provisionally accepted for publication in PLOS Computational Biology.

Before your manuscript can be formally accepted you will need to complete some formatting changes, which you will receive in a follow up email. A member of our team will be in touch with a set of requests.

Please note that your manuscript will not be scheduled for publication until you have made the required changes, so a swift response is appreciated.

IMPORTANT: The editorial review process is now complete. PLOS will only permit corrections to spelling, formatting or significant scientific errors from this point onwards. Requests for major changes, or any which affect the scientific understanding of your work, will cause delays to the publication date of your manuscript.

Should you, your institution's press office or the journal office choose to press release your paper, you will automatically be opted out of early publication. We ask that you notify us now if you or your institution is planning to press release the article. All press must be co-ordinated with PLOS.

Thank you again for supporting Open Access publishing; we are looking forward to publishing your work in PLOS Computational Biology.

Best regards,

Shaun Mahony

Section Editor

PLOS Computational Biology

***********************************************************

Reviewer's Responses to Questions

**Comments to the Authors:**

**Please note here if the review is uploaded as an attachment.**

Reviewer #1: I would like to thank the authors for their responses to my review. The authors have effectively and thoroughly addressed my points of feedback, and the manuscript is now significantly strengthened by several new analyses and textual corrections. I believe the paper is now appropriate for publication.

Reviewer #3: I find that Munn et al have done a reasonable amount of work to address the concerns of the reviewers. In fact, as someone who works in genome annotation, I'm still not entirely sure that the method will gain substantial traction in the field; not because it is not useful, rather because people will continue to use other more conventional approaches (i.e. regular transcriptomics). Instead, I find the real value here to be in the procedural work, which is interesting and technically sound, and leads to learning outcomes. As such, I think it will be especially interesting to those developing next-level computational approaches incorporating ML and CNN components.

**********

**Have the authors made all data and (if applicable) computational code underlying the findings in their manuscript fully available?**

The PLOS Data policy requires authors to make all data and code underlying the findings described in their manuscript fully available without restriction, with rare exception (please refer to the Data Availability Statement in the manuscript PDF file). The data and code should be provided as part of the manuscript or its supporting information, or deposited to a public repository. For example, in addition to summary statistics, the data points behind means, medians and variance measures should be available. If there are restrictions on publicly sharing data or code —e.g. participant privacy or use of data from a third party—those must be specified.

Reviewer #1: Yes

Reviewer #3: Yes

**********

PLOS authors have the option to publish the peer review history of their article (what does this mean?). If published, this will include your full peer review and any attached files.

If you choose “no”, your identity will remain anonymous but your review may still be made public.

**Do you want your identity to be public for this peer review?**  For information about this choice, including consent withdrawal, please see our Privacy Policy.

Reviewer #1: No

Reviewer #3: No

# Editor report: Acceptance letter

**DOI:** 10.1371/journal.pcbi.1014559.r004

**Authors:** Shaun Mahony

PCOMPBIOL-D-25-00811R1

Accurate de novo transcription unit annotation from run-on and sequencing data

Dear Dr Danko,

I am pleased to inform you that your manuscript has been formally accepted for publication in PLOS Computational Biology. Your manuscript is now with our production department and you will be notified of the publication date in due course.

The corresponding author will soon be receiving a typeset proof for review, to ensure errors have not been introduced during production. Please review the PDF proof of your manuscript carefully, as this is the last chance to correct any errors. Please note that major changes, or those which affect the scientific understanding of the work, will likely cause delays to the publication date of your manuscript.

Soon after your final files are uploaded, unless you have opted out, the early version of your manuscript will be published online. The date of the early version will be your article's publication date. The final article will be published to the same URL, and all versions of the paper will be accessible to readers.

For Research, Software, and Methods articles, you will receive an invoice from PLOS for your publication fee after your manuscript has reached the completed accept phase. If you receive an email requesting payment before acceptance or for any other service, this may be a phishing scheme. Learn how to identify phishing emails and protect your accounts at https://explore.plos.org/phishing.

Thank you again for supporting PLOS Computational Biology and open-access publishing. We are looking forward to publishing your work!

With kind regards,

Janani Seenivasan

PLOS Computational Biology | Carlyle House, Carlyle Road, Cambridge CB4 3DN | United Kingdom ploscompbiol@plos.org | Phone +44 (0) 1223-442824 | ploscompbiol.org | @PLOSCompBiol
